# Commensal oral microbiota induces osteoimmunomodulatory effects separate from systemic microbiome in mice

**DOI:** 10.1172/jci.insight.140738

**Published:** 2022-02-22

**Authors:** Jessica D. Hathaway-Schrader, Johannes D. Aartun, Nicole A. Poulides, Megan B. Kuhn, Blakely E. McCormick, Michael E. Chew, Emily Huang, Richard P. Darveau, Caroline Westwater, Chad M. Novince

**Affiliations:** 1 Department of Oral Health Sciences, College of Dental Medicine,; 2 Department of Pediatrics-Division of Endocrinology, College of Medicine, and; 3 Department of Stomatology-Division of Periodontics, College of Dental Medicine, Medical University of South Carolina (MUSC), Charleston, South Carolina, USA.; 4 Department of Periodontics, School of Dentistry, and; 5 Department of Oral Health Sciences, School of Dentistry, University of Washington, Seattle, Washington, USA.; 6 Department of Microbiology and Immunology, Hollings Cancer Center, MUSC, Charleston, South Carolina, USA.

**Keywords:** Bone Biology, Microbiology, Bone marrow, MHC class 2, Osteoclast/osteoblast biology

## Abstract

Commensal microbes critically regulate skeletal homeostasis, yet the impact of specific microbiota communities on osteoimmune response mechanisms is unknown. To discern osteoimmunomodulatory effects imparted by the commensal oral microbiota that are distinct from the systemic microbiota, osteoimmunology studies were performed in both alveolar bone and nonoral skeletal sites of specific pathogen–free (SPF) versus germ-free (GF) mice and SPF mice subjected to saline versus chlorhexidine oral rinses. SPF versus GF mice had reduced cortical/trabecular bone and an enhanced pro-osteoclastic phenotype in alveolar bone. TLR signaling and Th17 cells that have known pro-osteoclastic actions were increased in alveolar BM, but not long BM, of SPF versus GF mice. MHC II antigen presentation genes and activated DCs and CD4^+^ T cells were elevated in alveolar BM, but not long BM, of SPF versus GF mice. These findings were substantiated by in vitro allostimulation studies demonstrating increased activated DCs derived from alveolar BM, but not long BM, of SPF versus GF mice. Chlorhexidine antiseptic rinse depleted the oral, but not gut, bacteriome in SPF mice. Findings from saline- versus chlorhexidine-treated SPF mice corroborated outcomes from SPF versus GF mice, which reveals that the commensal oral microbiota imparts osteoimmunomodulatory effects separate from the systemic microbiome.

## Graphical abstract

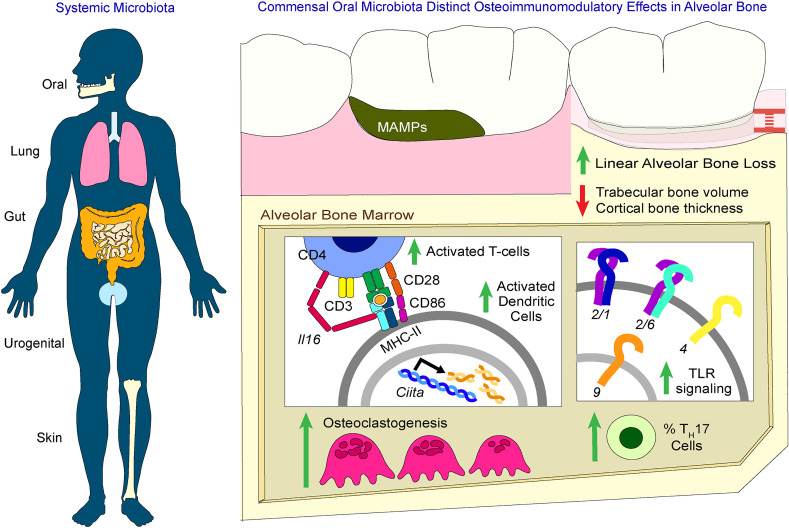

## Introduction

The host is colonized by diverse microorganisms at mucosal barrier surfaces exposed to the external environment. The collection of microbes colonizing distinct anatomic sites form unique microbial communities known as microbiota (i.e., oral, gut, skin, urogenital, lung) (1, 2). Early life host-microbe interactions direct the development of immunity and the establishment of a stable complex microbiota, which is referred to as the commensal (normal) microbiota (2, 3). Throughout the host’s life, immunity is stimulated by commensal microbiota–derived ligands that signal at pattern-recognition receptor–expressing (PRR-expressing) host cells. The host immune response maintains a homeostatic relationship with the commensal microbiota but, importantly, also has potent indirect effects on host physiology (4, 5).

The field of osteoimmunology has demonstrated that immune cell–bone cell interactions regulate bone metabolism and skeletal homeostasis (6–8). Proinflammatory immune mediators enhance osteoclastic-mediated bone resorption, which can have detrimental effects on the maintenance of bone mass in the adult skeleton (9–11). The commensal microbiota has recently been introduced as a critical regulator of physiologic osteoimmune processes in the healthy, adult skeleton, both at oral and nonoral skeletal sites (8, 12–14). However, the impact of specific commensal microbiota communities on osteoimmune response mechanisms is unknown.

The oral cavity is a distinct anatomic mucosal barrier site to colonizing microbes due to the presence of the teeth, a transmucosal organ that is integrated with alveolar bone. The commensal oral microbiota, which forms vigorous biofilms on the surfaces of the teeth, is separated from the underlying alveolar bone by epithelium and gingival connective tissue. The proximity of the commensal oral microbiota to alveolar bone is unique in that no other microbiota community (i.e., gut, skin, urogenital, lung) colonizes an external body surface integrated with osseous tissue (15).

Irie et al. performed the first known osteoimmunology study investigating the impact of the commensal oral microbiota on alveolar bone homeostasis (13). Young adult 11- to 12-week-old germ-free (GF) versus specific pathogen–free (SPF) mice were used to discern the commensal oral microbiota’s osteoimmunoregulatory effects on alveolar bone homeostasis. The burden of the commensal microbiota in SPF mice increased the osteoclastic cell numbers lining the alveolar bone crest (ABC) and exacerbated linear alveolar bone loss. Study outcomes demonstrating that SPF mice had increased neutrophils, CD3^+^ cells, CD4^+^ cells, and IL-17^+^ cells in the junctional epithelium led the authors to conclude that the commensal oral microbiota has immunostimulatory actions in the epithelial barrier tissues, which enhance osteoclastogenesis and drive alveolar bone loss in periodontal health (13).

The SPF versus GF mouse model has been used by our research group and others to discern the influence of the commensal oral microbiota on osteoimmune processes in alveolar bone (13, 16, 17). However, these published reports are confounded by 2 major limitations. The first limitation is that these prior studies relied on evaluating periodontal immune response mechanisms in barrier epithelial/gingival tissues and the underlying periosteal surface of the alveolar bone (13, 16, 17). Osteoimmune response mechanisms were not evaluated in the marrow space of the alveolar bone, which was due to the inability to isolate BM from the murine alveolar bone complex. The second limitation of these previous investigations was failure to recognize that microbiota differences in SPF versus GF mice are not limited to the presence/absence of the commensal oral microbiota (13, 16, 17). Whereas GF mice are devoid of microbes, SPF mice are colonized by the oral, gut, skin, urogenital, and lung commensal microbiota. Considering the different commensal microbiota communities all have immunoregulatory actions, which can modulate circulating immune factors (1, 2), the commensal microbiota collectively influences skeletal metabolism through endocrine signaling effects.

Potentially novel approaches and methodology were executed in the SPF versus GF mouse model to determine whether the commensal oral microbiota imparts osteoimmune response effects in alveolar bone that are separate from the systemic microbiome. We developed an approach for isolating BM cells from the murine mandible, which enabled us to perform advanced osteoimmunology studies in the alveolar bone complex. Relative comparison of alveolar bone alterations to nonoral skeletal site differences found in SPF versus GF mice facilitated elucidating that specific commensal microbiota communities have the capacity to impart distinct osteoimmunoregulatory effects.

Chlorhexidine gluconate (CHX; 0.12%) oral rinse is FDA approved as an anti-plaque agent for the treatment of gingivitis, and previous reports have demonstrated that CHX has protective effects in preclinical periodontitis-driven alveolar bone loss (18, 19). Furthermore, clinical CHX oral rinse studies have shown a significant reduction in oral bacterial load (20–22) and shifts in the healthy oral microbiome (23, 24), yet CHX has not been previously investigated as a therapeutic agent to support alveolar bone health and homeostasis. The 0.12% CHX oral rinse studies were carried out in SPF mice, and they discerned that local antiseptic depletion of the commensal oral microbiota protects against alveolar bone loss in the healthy periodontium. Study outcomes support the notion that the commensal oral microbiota imparts osteoimmune response effects that are separate from the systemic microbiome.

## Results

### Commensal microbiota has proinflammatory immunostimulatory effects in barrier periodontal tissues.

**Figure 1:**
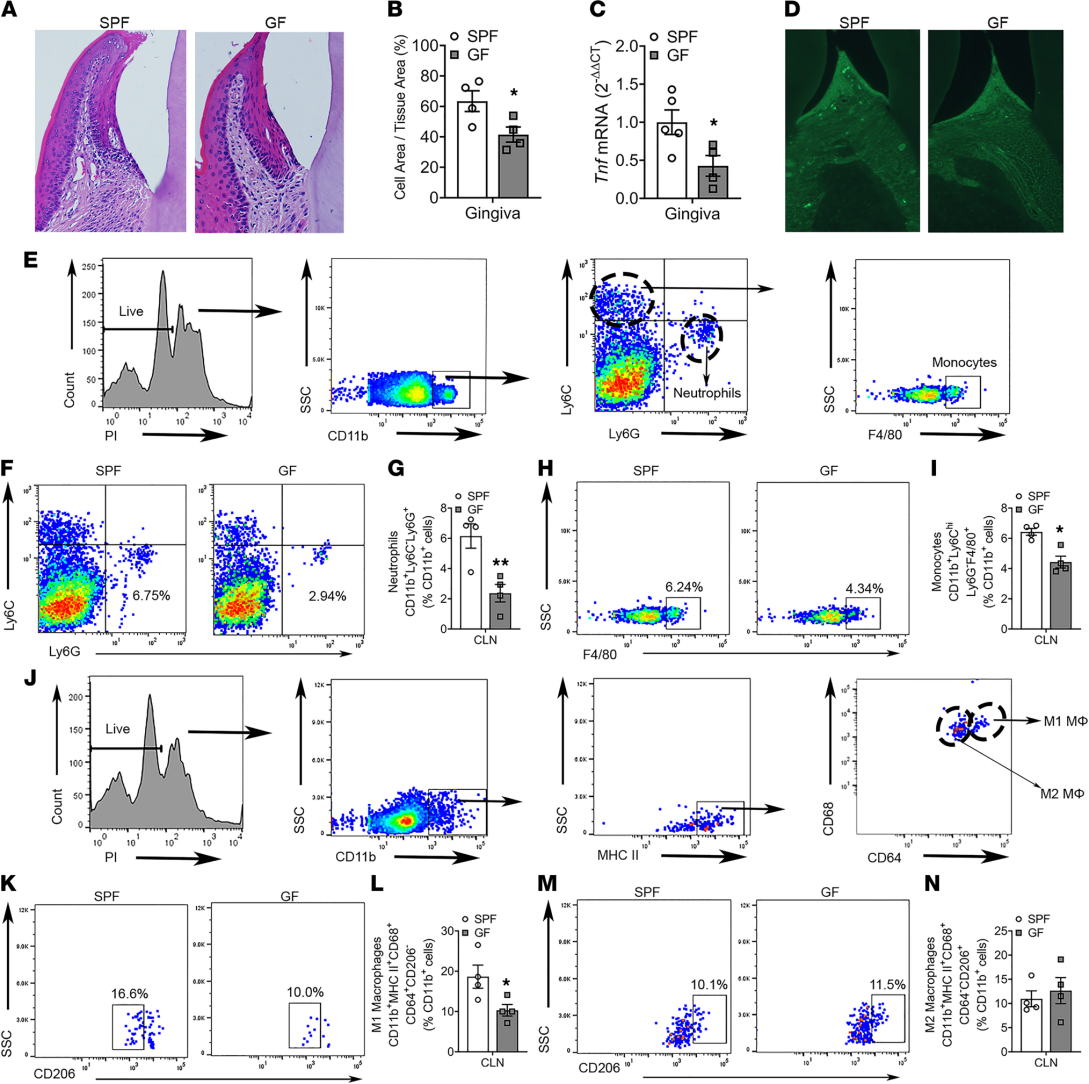
Commensal microbiota has proinflammatory immunostimulatory effects in barrier periodontal tissues. (**A**) Representative H&E-stained sections of junctional epithelium and gingival connective tissue at the buccal aspect of the mandibular first molar (×200). (**B**) Inflammatory cell infiltrate within junctional epithelium and gingival connective tissue of the mandibular first molar; cell area per tissue area (%); *n* = 4/gp. (**C**) qPCR analysis of *Tnf* mRNA in mandibular gingiva; *n* = 4–5/gp. (**D**) Sagittal sections of maxillae were stained with LYVE-1–FITC; representative immunofluorescence of LYVE-1 in the interdental space between first and second molars (×200). (**E**–**N**) Flow cytometric analysis evaluated proinflammatory innate immune cells in oral draining cervical lymph nodes (CLNs); *n* = 4/gp. (**E**) Representative gating strategy for neutrophils and monocytes. Representative dot plots and quantitation for (**F** and **G**) CD11b^+^Ly6C^–^Ly6G^+^ neutrophils and (**H** and **I**) CD11b^+^Ly6C^hi^Ly6G^–^F4/80^+^ inflammatory monocytes; reported as percentage of CD11b^+^ cells. (**J**) Representative gating strategy for M1 and M2 macrophages. Representative dot plots and quantitation for (**K** and **L**) CD11b^+^MHC II^+^CD68^+^CD64^+^CD206^–^ M1 macrophages and (**M** and **N**) CD11b^+^MHC II^+^CD68^+^CD64^–^CD206^+^ M2 macrophages; reported as percentage of CD11b^+^ cells. Unpaired *t* test; data presented as mean ± SEM; *P < 0.05, **P < 0.01.

The periodontal epithelium and gingival connective tissue act as physical barriers separating the oral microbiota from the ABC. Different from other epithelial tissues that act as an impermeable barrier to colonizing microbes, the junctional epithelium is highly permeable (25–27). Extensive prior research has delineated that microbial byproducts pass through the highly permeable junctional epithelium in both health and disease (28–34). SPF versus GF mice had increased inflammatory cell infiltrate (Figure 1, A and B) and proinflammatory cytokine gene expression (Figure 1C) in periodontal epithelium and gingival connective tissue.

Research has demonstrated that lymphatic vessels are a normal constituent of the healthy periodontium. The periodontal lymphatic vasculature extends from the junctional epithelium, through the gingival connective tissue, and penetrates the alveolar BM (35–38). To assess periodontal lymphatics, immunofluorescent labeling of lymphatic vessel endothelial receptor 1 (LYVE-1) was carried out in maxilla sections of SPF versus GF mice (Figure 1D). LYVE-1 expression was detected in the interdental gingival connective tissue of SPF versus GF mice (Figure 1D). To validate that the commensal oral microbiota has immunostimulatory effects in periodontal barrier tissues, oral draining cervical lymph nodes (CLNs) were isolated for flow cytometric analysis. The CLNs of SPF versus GF mice had increased frequencies of neutrophil cells (Figure 1, E–G), inflammatory monocytes (Figure 1, E, H, and I), and proinflammatory M1-macrophages (Figure 1, J–L).

### Burden of commensal oral microbiota drives alveolar bone loss.

**Figure 2:**
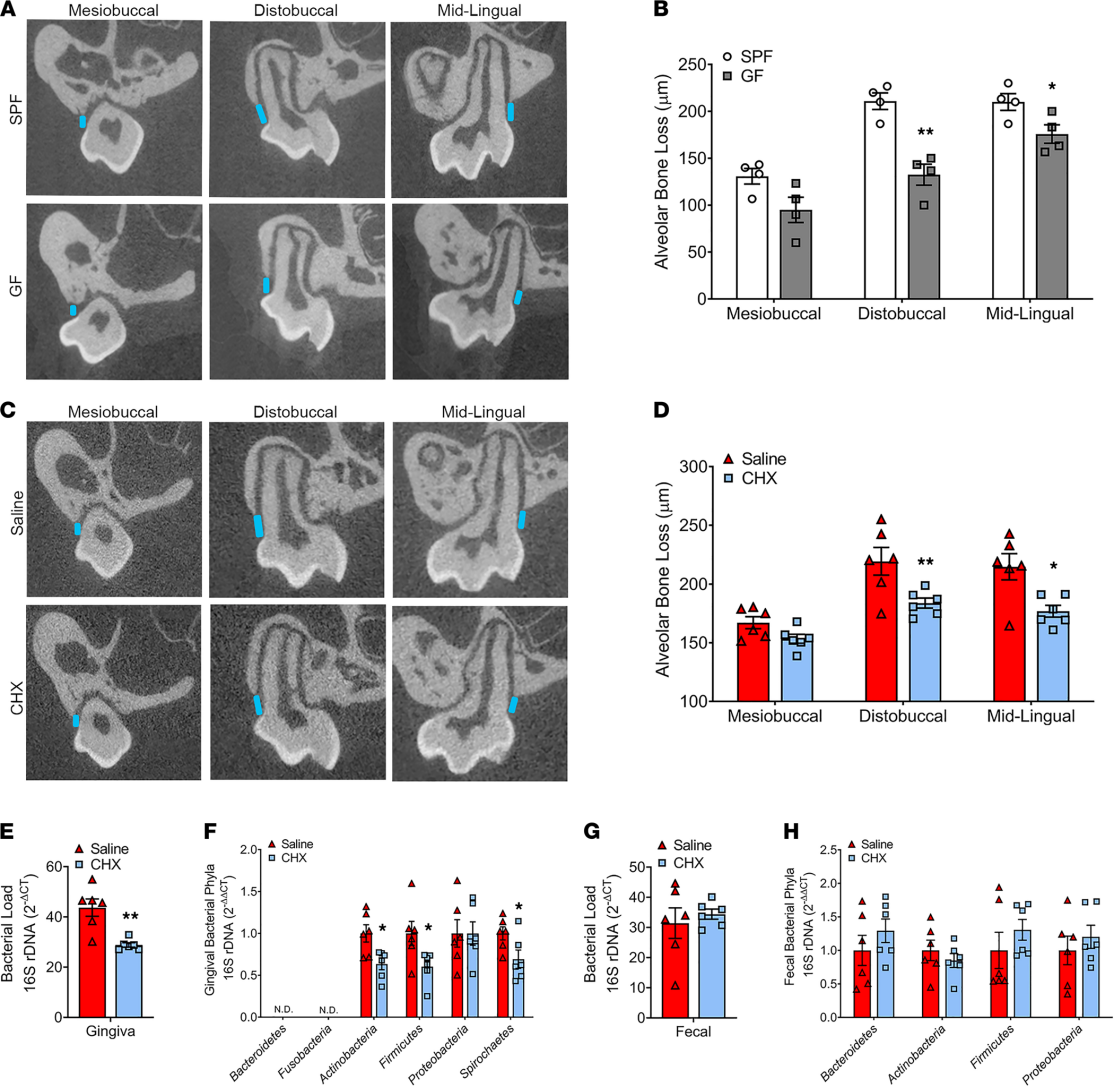
Burden of commensal oral microbiota drives alveolar bone loss. (**A**–**D**) Alveolar bone loss was assessed by evaluating the linear distance between the cementoenamel junction (CEJ) and alveolar bone crest (ABC) at the maxillary first molar in reconstructed μCT images. (**A** and **B**) Representative μCT images and quantitative measures of CEJ-ABC linear distance (blue line) at the mesiobuccal, distobuccal, and midlingual aspect of the maxillary first molar of SPF versus GF mice; *n* = 4/gp. (**C** and **D**) Representative μCT images and quantitative measures of CEJ-ABC linear distance at the mesiobuccal, distobuccal, and midlingual aspect of the maxillary first molar of SPF mice treated with saline or CHX oral rinse from age 6 to 12 weeks; *n* = 6/gp. (**E**–**H**) 16S rDNA analysis of bacterial load (reported as 2^–ΔCt^) and phylum levels (reported as 2^–ΔΔCt^) for (**E** and **F**) maxillary gingiva and (**G** and **H**) fecal pellets in SPF mice treated with saline or CHX oral rinse from age 6 to 12 weeks; *n* = 6/gp. Unpaired *t* test; data are presented as mean ± SEM; *P < 0.05, **P < 0.01.

Alveolar bone loss is experimentally and clinically assessed by measuring the linear distance from the cementoenamel junction (CEJ) to the ABC (39). Morphometric analysis of CEJ-ABC linear distance was performed at 3 clinically relevant sites in reconstructed μCT images of the maxillary first molar (Figure 2A). SPF versus GF mice had significantly greater linear alveolar bone loss at the distobuccal line angle and mid-lingual aspect of the maxillary first molar (Figure 2, A and B), which is consistent with prior investigations (13, 40–42).

The upregulated proinflammatory innate immune response in barrier periodontal tissues (Figure 1) and greater linear alveolar bone loss found in SPF versus GF mice (Figure 2, A and B) implies that the commensal oral microbiota induces osteoimmunomodulatory effects on the proximal alveolar bone. Appreciating that SPF mice are colonized by diverse commensal microbiota communities (i.e., oral, gut, skin, urogenital, lung) that constitute the systemic microbiome, alveolar bone differences observed when comparing SPF to GF mice cannot be solely attributed to the commensal oral microbiota. To delineate whether the commensal oral microbiota imparts osteoimmune response effects that are separate from the systemic microbiome, an experimental 0.12% CHX oral rinse model was employed, and osteoimmunology investigations were carried out at oral and nonoral skeletal sites.

### Antiseptic depletion of the commensal oral microbiota protects against alveolar bone loss in the healthy periodontium.

**Figure 3:**
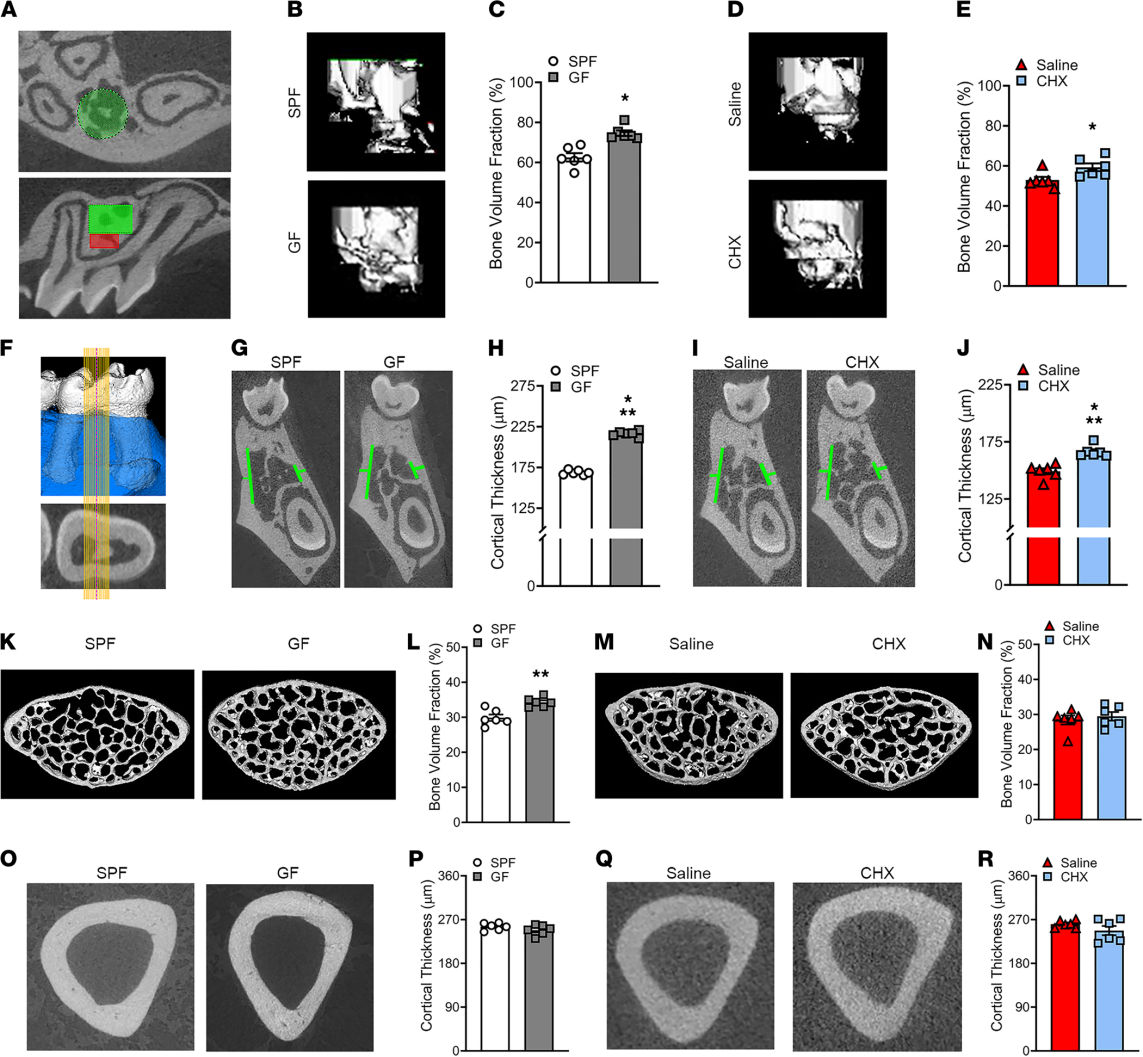
Commensal oral microbiota has catabolic effects on alveolar bone microarchitecture, which are distinct from systemic microbiota effects on the nonoral skeletal sites. (**A**) μCT images displaying the trabecular bone region of interest (ROI; red and green cylinders) within the maxillary first molar furcation. (**B** and **C**) Representative μCT images and quantitative analysis of trabecular bone volume fraction (%) in maxillary first molar furcation of SPF versus GF mice; *n* = 6/gp. (**D** and **E**) Representative μCT images and quantitative analysis of trabecular bone volume fraction (%) in maxillary first molar furcation of saline versus CHX mice; *n* = 6/gp. (**F**) Cortical thickness was evaluated in a 200 μm mesial-distal ROI in the mandibular first molar bifurcation. ROI centered at the midpoint between the mesial and distal roots; 10 measures analyzed mesial and distal to midpoint. (**G** and **H**) Representative μCT images and quantitative analyses of cortical bone thickness (green lines) in the mandibular first molar furcation of SPF versus GF mice; *n* = 6/gp. (**I** and **J**) Representative μCT images and quantitative analyses of cortical bone thickness in the mandibular first molar furcation of saline versus CHX mice; *n* = 6/gp. (**K** and **L**) Representative μCT images and quantitative analysis of fifth lumbar vertebral trabecular bone volume fraction (%) in SPF versus GF mice; *n* = 6/gp. (**M** and **N**) Representative μCT images and quantitative analysis of fifth lumbar vertebral trabecular bone volume fraction (%) in saline versus CHX mice; *n* = 6/gp. (**O** and **P**) Representative μCT images and quantitative analyses of cortical bone thickness in the tibia mid-diaphysis of SPF versus GF mice; *n* = 6/gp. (**Q** and **R**) Representative μCT images and quantitative analyses of cortical bone thickness in the tibia mid-diaphysis of saline versus CHX mice; *n* = 6/gp. Unpaired *t* test; data are presented as mean ± SEM; *P < 0.05, **P < 0.01, ***P < 0.001.

**Figure 4:**
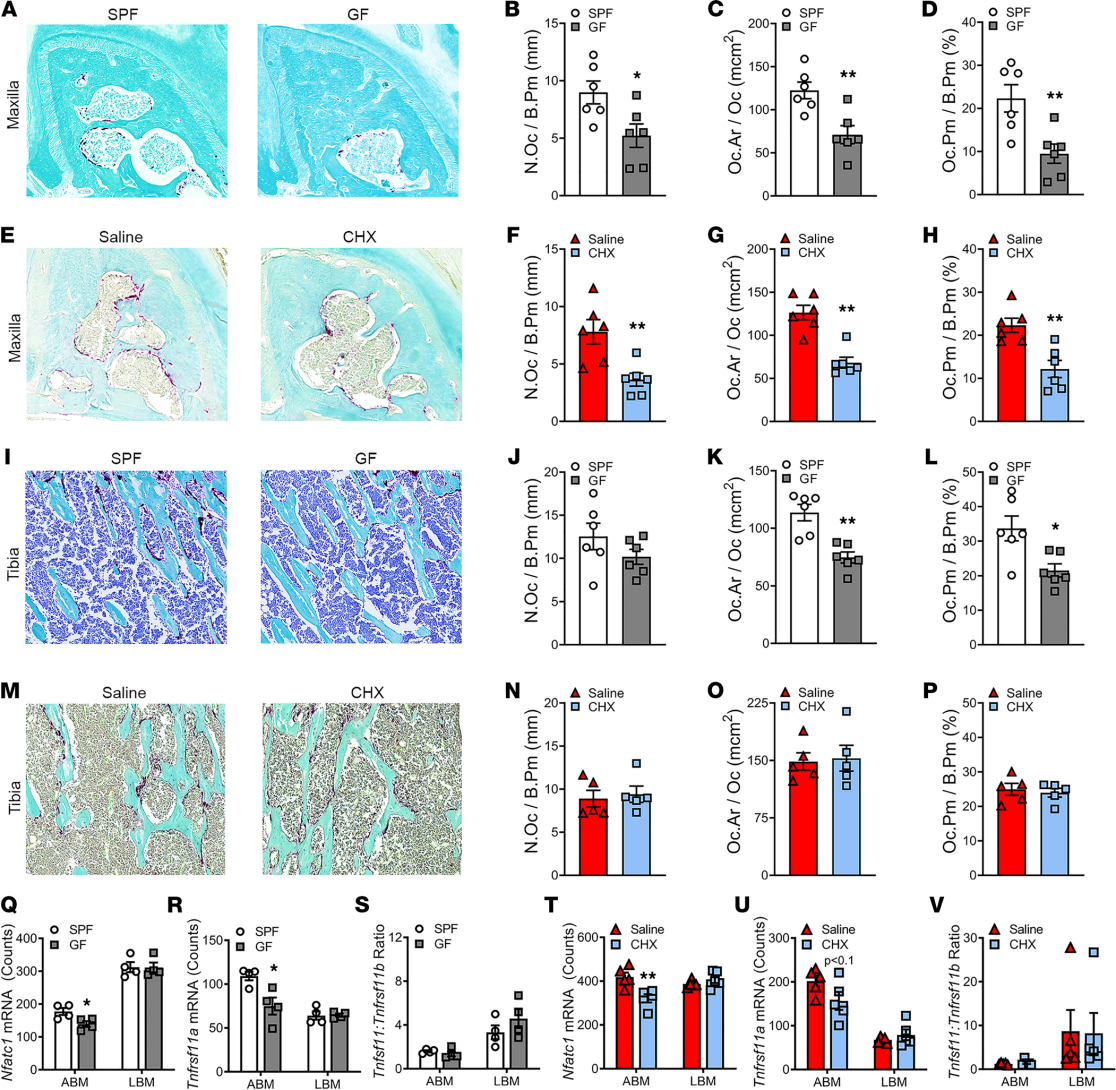
Commensal oral microbiota has pro-osteoclastic actions distinct from the systemic microbiota, which enhance osteoclast numbers lining alveolar BM. (**A**–**H**) Sagittal sections of the maxillary first molar were TRAP stained for osteoclast cellular outcomes in (**A**–**D**) SPF versus GF mice (*n* = 6/gp) and (**E**–**H**) saline versus CHX mice (*n* = 6/gp). (**A**) Representative TRAP stain in the maxillary first molar furcation of SPF versus GF mice (×200). (**E**) Representative TRAP stain in the maxillary first molar furcation of saline versus CHX mice (×200). (**I**–**P**) Frontal sections of proximal tibia TRAP stained for osteoclast cellular outcomes in (**I**–**L**) SPF versus GF mice (*n* = 6/gp) and (**M**–**P**) saline versus CHX mice (*n* = 6/gp). (**I**) Representative TRAP stain in proximal tibia of SPF versus GF mice (×200). (**M**) Representative TRAP stain in proximal tibia of saline versus CHX mice (×200). (**Q**–**S**) nCounter analysis of osteoclast genes and signaling factors in the alveolar BM (ABM) and long BM (LBM) of (**Q**–**S**) SPF versus GF mice (*n* = 4/gp). (**T**–**V**) nCounter analysis of osteoclast genes and signaling factors in the ABM and LBM of saline versus CHX mice (*n* = 6/gp). Unpaired *t* test; data reported as mean ± SEM; *P < 0.05, **P < 0.01.

**Figure 5:**
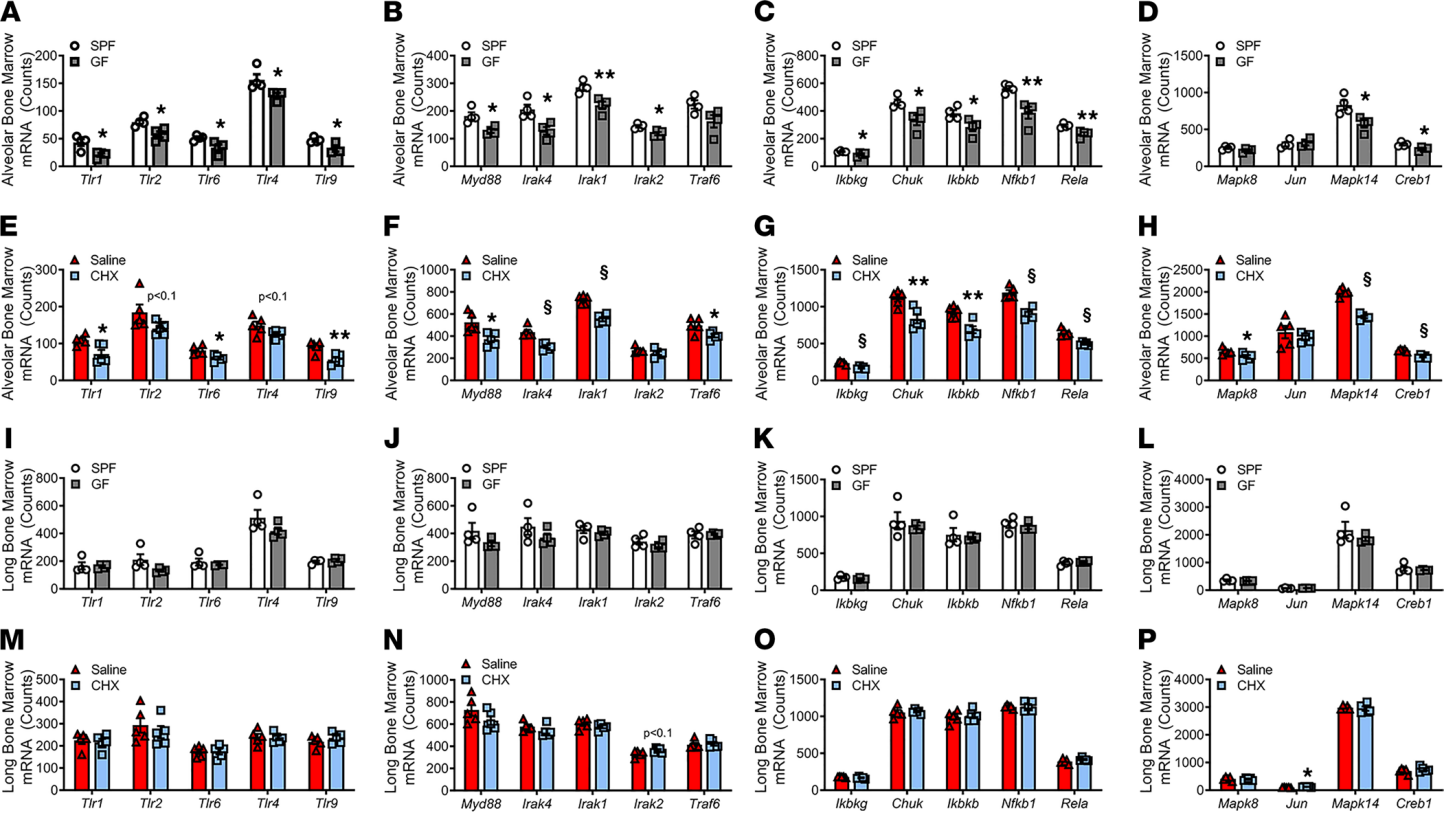
Commensal oral microbiota has immunostimulatory actions distinct from the systemic microbiota, which substantially upregulates TLR signaling in alveolar BM. (**A**–**D**) nCounter analysis was utilized to determine the expression of TLRs and related downstream signaling molecules in the alveolar BM (ABM) of SPF versus GF mice; *n* = 4/gp. (**A**) TLR expression. (**B**) MyD88-dependent adaptor molecule expression. (**C**) NF‑κB signaling factor expression. (**D**) MAPK signaling factor expression. (**E**–**H**) nCounter analysis of TLRs and related downstream signaling molecules in the ABM of saline versus CHX mice; *n* = 6/gp. (**E**) TLR expression. (**F**) MyD88-dependent adaptor molecule expression. (**G**) NF‑κB signaling factor expression. (**H**) MAPK signaling factor expression. (**I**–**L**) nCounter analysis of TLRs and related downstream signaling molecules in the long BM (LBM) of SPF versus GF mice; *n* = 4/gp. (**I**) TLR expression. (**J**) MyD88-dependent adaptor molecule expression. (**K**) NF‑κB signaling factor expression. (**L**) MAPK signaling factor expression. (**M**–**P**) nCounter analysis TLRs and related downstream signaling molecules in the LBM of saline versus CHX mice; *n* = 6/gp. (**M**) TLR expression. (**N**) MyD88-dependent adaptor molecule expression. (**O**) NF‑κB signaling factor expression. (**P**) MAPK signaling factor expression. Unpaired *t* test; data presented as mean ± SEM; *P < 0.05, **P < 0.01, ^§^*P* < 0.001.

**Figure 6:**
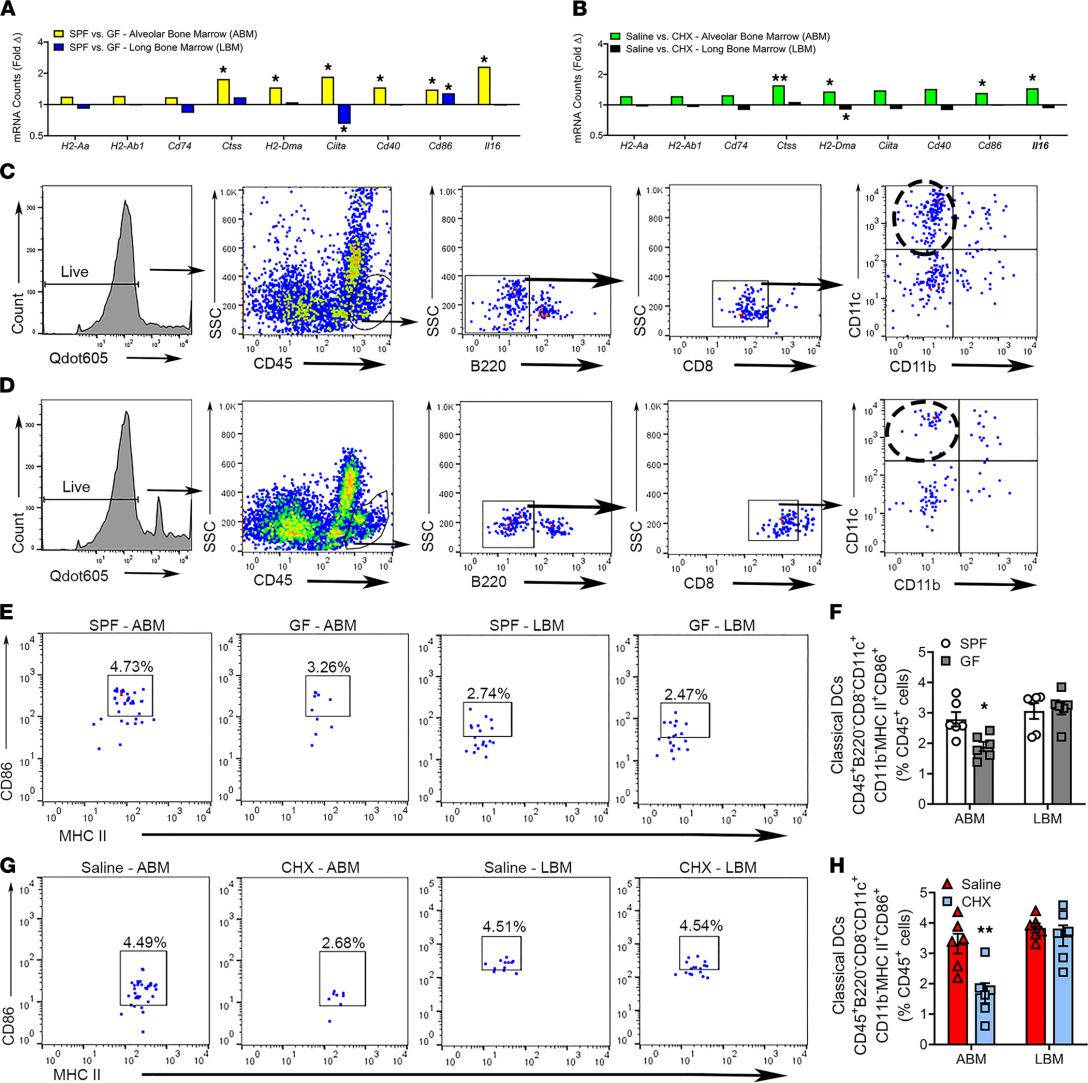
Commensal oral microbiota supports DC upregulation through MHC II–associated genes in alveolar BM. (**A** and **B**) nCounter analysis was performed to assess MHC II antigen processing and presentation genes in the alveolar BM (ABM) and long BM (LBM) of (**A**) SPF versus GF mice (*n* = 4/gp) and (**B**) saline versus CHX mice (*n* = 6/gp). Data reported as fold change difference. (**C** and **D**) Representative gating strategy for classical dendritic cells in (**C**) ABM and (**D**) LBM. (**E** and **F**) Representative dot plots and quantitative measures for CD45^+^B220^–^CD8^–^CD11c^+^CD11b^–^MHC II^+^CD86^+^ classical DCs in ABM and LBM of SPF versus GF mice; reported as percentage of CD45^+^ cells. (**G** and **H**) Representative dot plots of final gating and quantitative measures for CD45^+^B220^–^CD8^–^CD11c^+^CD11b^–^MHC II^+^CD86^+^ classical dendritic cells in ABM and LBM of saline versus CHX mice; reported as percentage of CD45^+^ cells. Unpaired *t* test; data presented as mean ± SEM; *P < 0.05, **P < 0.01.

**Figure 7:**
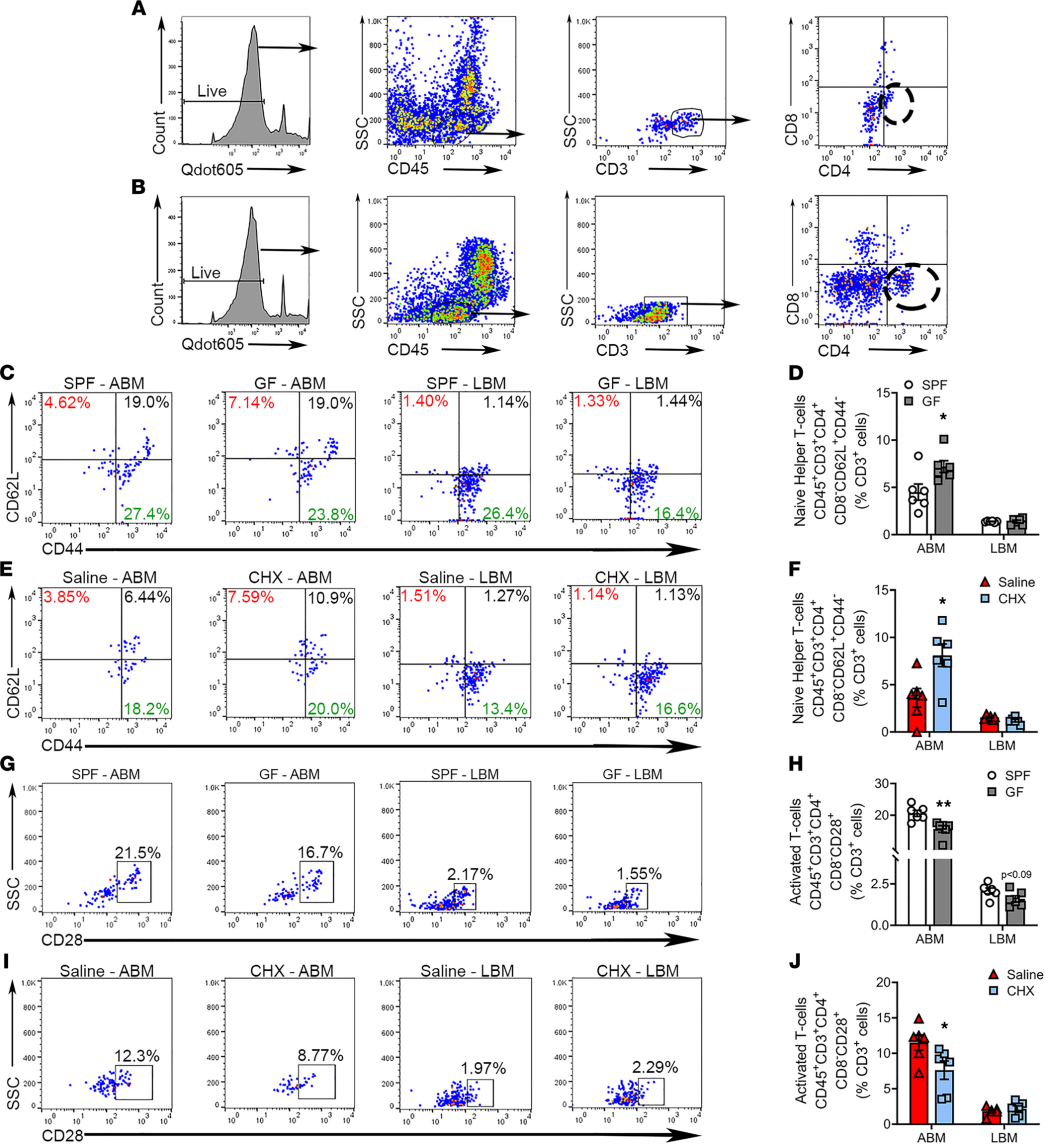
Commensal oral microbiota promotes Th cell activation in alveolar BM. Flow cytometric analysis of CD4^+^ Th cells in the alveolar BM (ABM) and long BM (LBM), reported as percentage of CD3^+^ cells; *n* = 6/gp. (**A** and **B**) Representative gating strategy for CD4^+^ T cells in (**A**) ABM and (**B**) LBM. (**C** and **D**) Representative dot plots and quantitation (upper left; red %) for CD45^+^CD3^+^CD4^+^CD8^–^CD62L^+^CD44^–^ naive CD4^+^ T cells in ABM and LBM of SPF versus GF mice. (**E** and **F**) Representative dot plots and quantitation (upper left; red %) for CD45^+^CD3^+^CD4^+^CD8^–^CD62L^+^CD44^–^ naive CD4^+^ T cells in ABM and LBM of saline versus CHX mice. (**G** and **H**) Representative dot plots and quantitation for CD45^+^CD3^+^CD4^+^CD8^–^CD28^+^ activated CD4^+^ T cells in ABM and LBM of SPF versus GF mice. (**I** and **J**) Representative dot plots and quantitation for CD45^+^CD3^+^CD4^+^CD8^–^CD28^+^ activated CD4^+^ T cells in ABM and LBM of saline versus CHX mice. Unpaired *t* test; data presented as mean ± SEM; *P < 0.05, **P < 0.01.

**Figure 8:**
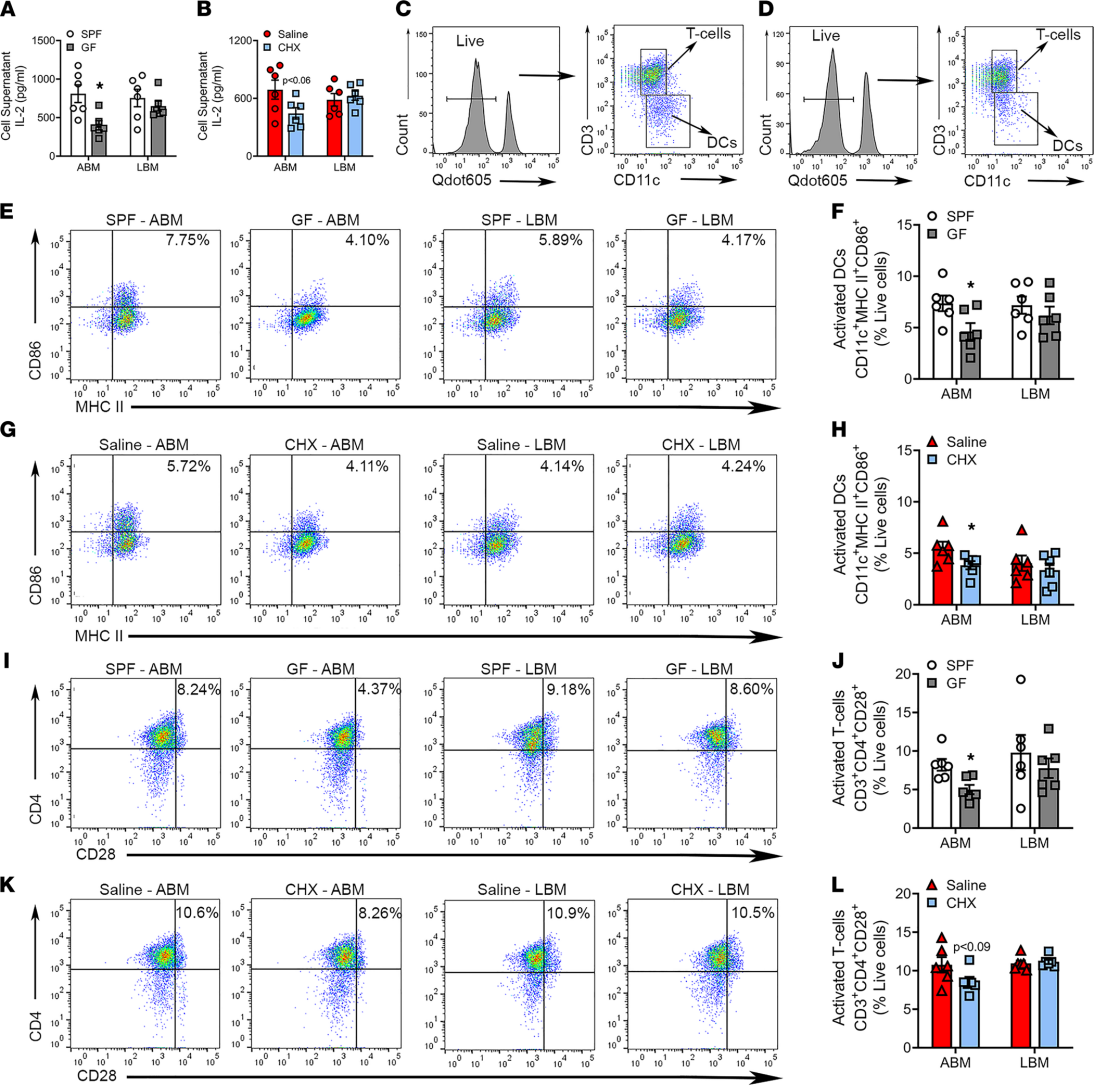
Commensal oral microbiota promotes activation of alveolar BM–derived DCs in vitro. (**A** and **B**) DC/T cell allostimulation assay supernatants were collected for ELISA IL-2 analysis from (**A**) SPF versus GF alveolar BM (ABM) and long BM (LBM) cultures and (**B**) saline versus CHX ABM and LBM cultures. (**C** and **D**) Representative gating strategy for T cells and dendritic cells from (**C**) ABM and (**D**) LBM cultures. (**E** and **F**) Representative dot plots of final gating and quantitative measures for CD11c^+^MHC II^+^CD86^+^ activated DCs from ABM and LBM cultures derived from SPF versus GF mice; reported as percentage of live cells. (**G** and **H**) Representative dot plots of final gating and quantitative measures for CD11c^+^MHC II^+^CD86^+^ activated DCs in ABM and LBM cultures derived from saline versus CHX mice; reported as percentage of live cells. (**I** and **J**) Representative dot plots of final gating and quantitative measures for CD3^+^CD4^+^CD28^+^ activated T cells in ABM and LBM cultures derived from SPF versus GF mice; reported as percentage of live cells. (**K** and **L**) Representative dot plots of final gating and quantitative measures for CD3^+^CD4^+^CD28^+^ activated T cells in ABM and LBM cultures derived from saline versus CHX mice; reported as percentage of live cells. Unpaired *t* test; data presented as mean ± SEM; *P < 0.05.

**Figure 9:**
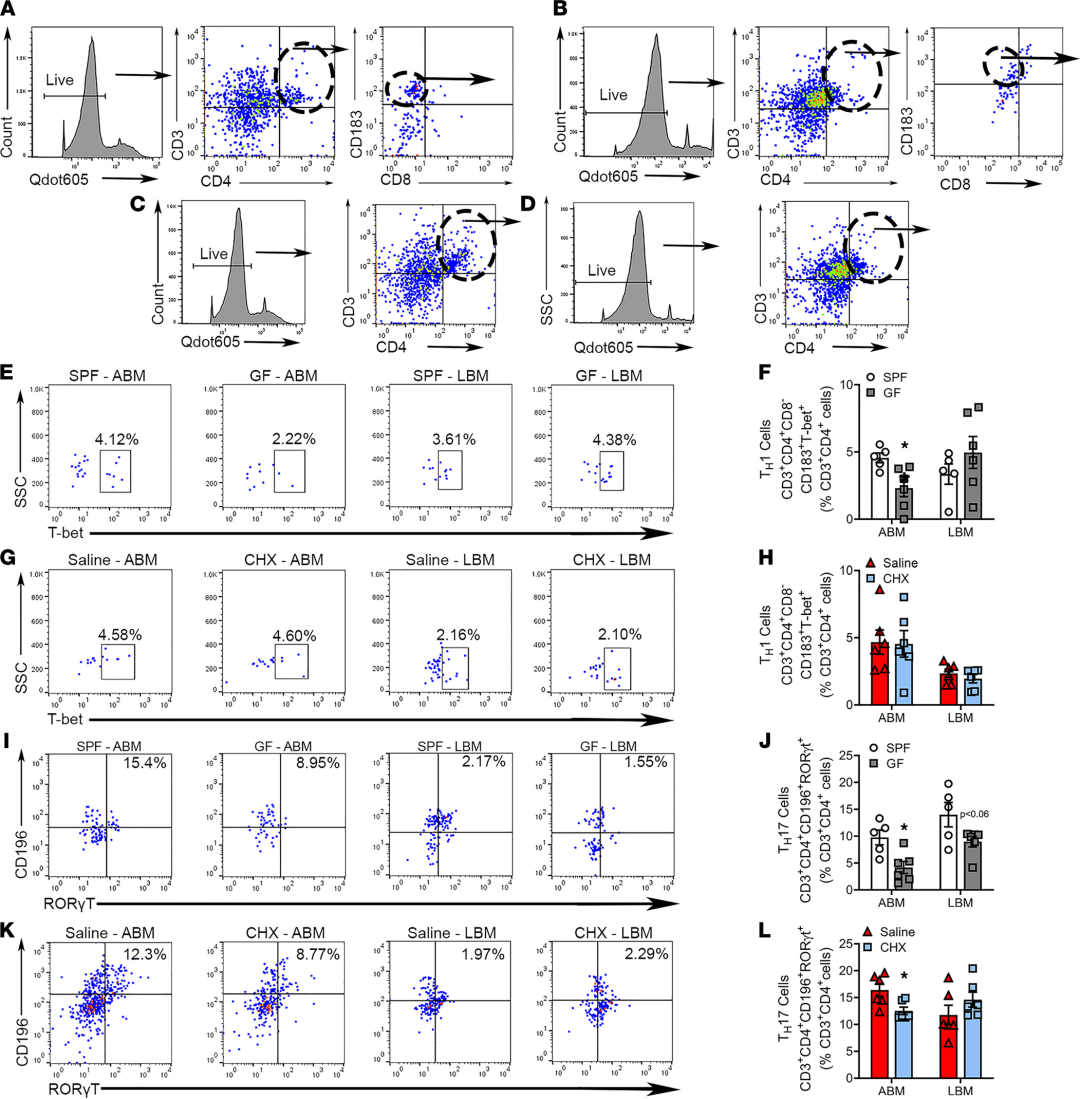
Commensal oral microbiota upregulates proinflammatory CD4^+^ T cell–mediated immunity in alveolar BM. Flow cytometric analysis of CD4^+^ Th cell subsets in the alveolar BM (ABM) and long BM (LBM), reported as percentage of CD3^+^CD4^+^ cells; *n* = 5–6/gp. (**A** and **B**) Representative gating strategy for Th1 cells in (**A**) ABM and (**B**) LBM. (**C** and **D**) Representative gating strategy for Th17 cells in (**C**) ABM and (**D**) LBM. (**E** and **F**) Representative dot plots of final gating and quantitative measures for CD3^+^CD4^+^CD8^–^CD183^+^T-bet^+^ Th1 cells in MM and LBM of SPF versus GF mice. (**G** and **H**) Representative dot plots of final gating and quantitative measures for CD3^+^CD4^+^CD8^–^CD183^+^T-bet^+^ Th1 cells in ABM and LBM of saline versus CHX mice. (**I** and **J**) Representative dot plots of final gating and quantitative measures for CD3^+^CD4^+^CD196^+^RORγt^+^ Th17 cells in ABM and LBM of SPF versus GF mice. (**K** and **L**) Representative dot plots of final gating and quantitative measures for CD3^+^CD4^+^CD196^+^RORγt^+^ Th17 cells in ABM and LBM of saline versus CHX mice. Unpaired *t* test; data presented as mean ± SEM; *P < 0.05.

SPF mice were treated with saline-control or 0.12% CHX oral antiseptic rinse from age 8 to 12 weeks (Supplemental Figure 1; supplemental material available online with this article; https://doi.org/10.1172/jci.insight.140738DS1) and age 6 to 12 weeks (Figure 2, Figure 3, Figure 4, Figure 5, Figure 6, Figure 7, Figure 8, and Figure 9). The CHX oral rinses were carried out based on FDA-approved clinical guidelines for the treatment of gingivitis. Oral rinses were performed every 12 hours, using 150 μL volume for a duration of 30 seconds (Supplemental Video 1). Animals were euthanized 12 hours following the final oral rinse.

Paralleling the increased alveolar bone loss observed in SPF versus GF mice (Figure 2, A and B), SPF mice treated with saline versus CHX had greater linear alveolar bone loss at the maxillary first molar (Supplemental Figure 1 and Figure 2, C and D). The reduced alveolar bone loss in SPF mice treated with CHX rinse from age 8 to 12 weeks and age 6 to 12 weeks appears to be attributed to CHX antiseptic actions suppressing the oral bacterial load (Supplemental Figure 1B and Figure 2E). Alveolar bone formation is dependent on the development and eruption of the teeth (43, 44), a developmental process that is typically complete by age 5 weeks in mice (44–47). To optimize CHX rinse actions preventing bone loss in the mature alveolar bone complex, subsequent studies relied on treating SPF mice with saline versus CHX rinse from age 6 to 12 weeks (Figure 2, Figure 3, Figure 4, Figure 5, Figure 6, Figure 7, Figure 8, Figure 9).

Clinical research supports the notion that changes in the composition of the oral bacteriome contribute to host immune response effects that cause alveolar bone loss (48, 49). Therefore, 16S rDNA analysis was performed to evaluate bacterial phyla level alterations in the oral microbiome of saline versus CHX mice. CHX rinse treatment decreased the prominence of Actinobacteria, Firmicutes, and Spirochaetes but did not alter Proteobacteria (Figure 2F). The murine oral microbiome has just recently begun to be characterized in both health and disease (50, 51), and further research is needed to determine how shifts in the indigenous murine oral microbiota impact alveolar bone homeostasis.

16S investigations in fecal pellets showed that CHX rinse treatment did not alter the bacterial load (Supplemental Figure 1C and Figure 2G) or induce bacterial phyla level alterations (Figure 2H) in the gut microbiome. These results suggest that the antiseptic actions of CHX rinse are limited to the oral cavity. Notably, CHX study outcomes show that local antiseptic depletion of the commensal oral microbiota protects against alveolar bone loss in the healthy periodontium.

### Commensal oral microbiota has catabolic effects on alveolar bone microarchitecture, which are distinct from systemic microbiome effects on nonoral skeletal sites.

μCT morphometric analysis was carried out to evaluate trabecular and cortical bone parameters at oral and nonoral skeletal sites of SPF versus GF mice and saline versus CHX mice (Figure 3). Trabecular bone volume fraction was reduced in the maxillary first molar trifurcation (Figure 3A) of SPF versus GF mice (Figure 3, B and C) and saline versus CHX mice (Figure 3, D and E). Cortical thickness was decreased in the mandibular first molar bifurcation (Figure 3F) of SPF versus GF mice (Figure 3, G and H) and saline versus CHX mice (Figure 3, I and J). μCT studies at nonoral skeletal sites revealed that trabecular bone fraction was blunted in the L5 vertebral body of SPF versus GF mice (Figure 3, K and L) but not in saline versus CHX mice (Figure 3, M and N). There were no differences in the cortical thickness at the tibia mid-diaphysis of SPF versus GF mice (Figure 3, O and P) or saline versus CHX mice (Figure 3, Q and R).

### Commensal oral microbiota has pro-osteoclastic actions distinct from the systemic microbiota.

Histomorphometric analyses of tartrate-resistant acid phosphatase–stained (TRAP-stained) tissue sections was carried out to evaluate osteoclastic cells lining trabecular bone of SPF versus GF mice and saline versus CHX mice. The maxillary first molar trifurcation was chosen for alveolar bone analysis (Figure 4, A–H), and the proximal tibia was selected for nonoral skeletal site analysis (Figure 4, I–P). Osteoclast numbers lining bone (N.Oc/B.Pm) were increased and average osteoclast cell size (Oc.Ar/Oc) was larger in the alveolar bone of SPF versus GF mice (Figure 4, A–C) and saline versus CHX mice (Figure 4, E–G). Both the increased N.Oc/B.Pm and larger Oc.Ar/Oc contributed to an enhanced osteoclast perimeter per bone perimeter (Oc.Pm/B.Pm) in the alveolar bone of SPF versus GF mice (Figure 4D) and saline versus CHX mice (Figure 4H). N.Oc/B.Pm were not different in the proximal tibia of SPF versus GF mice (Figure 4, I and J) or saline versus CHX mice (Figure 4, M and N). Oc.Ar/Oc was larger (Figure 4K) in SPF versus GF mice, which resulted in a greater Oc.Pm/B.Pm (Figure 4L) in the proximal tibia of SPF mice. There were no differences in Oc.Ar/Oc and Oc.Pm/B.Pm (Figure 4, O and P) in the proximal tibia of saline versus CHX mice.

Gene expression studies were performed evaluating critical osteoclastic genes and signaling factors in BM (Figure 4, Q–V) to further delineate whether the commensal oral microbiota imparts pro-osteoclastic actions that are distinct from the systemic microbiome. In line with the increased osteoclast numbers lining the trabecular bone in the alveolar bone complex, *Nfatc1* (the master transcription factor for osteoclastogenesis) and *Tnfrsf11a* (*Rank*; the RANKL receptor and a surrogate marker for commitment to the preosteoclastic/osteoclastic cell lineage) were upregulated in the alveolar BM, but not the long BM, of SPF versus GF mice (Figure 4, Q and R) and saline versus CHX mice (Figure 4, T and U). Recognizing that RANKL binding at the RANK receptor is critical and necessary for osteoclastogenesis, differences were ruled out in the *Tnfsf11* (*Rankl*)*/Tnfrsf11b* (*Opg*) ratio (Figure 4, S and V).

### Commensal oral microbiota has immunostimulatory actions distinct from the systemic microbiota, which substantially upregulates TLR signaling in alveolar BM.

The seminal work by Rakoff-Nahoum et al. discerned microbial ligand signaling at TLRs is not unique to pathogenic microorganisms but, rather, includes microbial-associated molecular patterns (MAMPs) derived from both commensal and pathogenic microbes (52). Of interest, commensal microbiota–derived MAMPs have been reported to pass through epithelial barriers at low levels in health, which has implications for modulating hematopoiesis in the BM environment (1, 53–55). To discern whether commensal microbiota–derived MAMPs regulate TLR signaling in BM, the expression of TLRs and critical downstream signal transduction factors were evaluated by nCounter analysis (Figure 5). We focused our analysis on TLR family members that have been implicated in periodontitis-induced alveolar bone destruction (56–58).

TLR2 heterodimer components (*Tlr1*, *Tlr2*, *Tlr6*), *Tlr4*, and *Tlr9* were upregulated in the alveolar BM of SPF versus GF mice (Figure 5A). There were trends toward increased *Tlr2* and *Tlr4* while *Tlr1*, *Tlr6*, and *Tlr9* were significantly enhanced in saline versus CHX alveolar BM (Figure 5E). TLR-1/2/4/6/9 signaling is dependent on the myeloid differentiation primary-response protein 88 (MyD88) adapter protein (59, 60). TLR engagement of the MYD88 adapter protein initiates signal transduction that relies upon interactions between the adaptor molecules, IL‑1R–associated kinase 4 (IRAK4), IRAK1, IRAK2, and TNF receptor-associated factor 6 (TRAF6) (59, 60). *Myd88* and related adaptor molecules were upregulated in the alveolar BM of SPF versus GF mice (Figure 5B) and saline versus CHX mice (Figure 5F). MYD88-dependent downstream activation of mitogen-activated protein kinase (MAPK) and NF‑κB signaling ultimately leads to proinflammatory immune response effects (59, 60). Alveolar BM of SPF versus GF mice and saline versus CHX mice demonstrated increased NF‑κB (Figure 5, C and G) and MAPK (Figure 5, D and H) signal transduction. To the contrary, TLR expression, MYD88 and related adaptor molecules, NF‑κB, and MAPK signaling elements were similar within the long BM of SPF versus GF mice (Figure 5, I–L) and saline versus CHX mice (Figure 5, M–P). This investigation of TLR signaling in the BM at oral and nonoral skeletal sites intriguingly reveals that the commensal oral microbiota has unique immunoregulatory effects that drive the induction of MYD88-dependent TLR-mediated immunity in alveolar BM. Whereas experimental murine periodontitis investigations have shown that periopathogenic bacteria induced alveolar bone destruction is mediated in part by costimulation of TLR2, TLR4, and TLR9 (56–58), outcomes from the current report imply that the commensal oral microbiota–driven alveolar bone loss is governed by TLR2, TLR4, and TLR9 signaling crosstalk.

### Commensal oral microbiota supports DC upregulation through MHC II–associated genes in alveolar BM.

Reports have shown that TLR and MHC II antigen presentation pathways can promote one another, due to similar processing of ligands/antigens within endosomal cellular compartments (61–63). MHC II antigen processing and presentation are critical for professional antigen-presenting cells, such as B cells, macrophages, and DCs, to determine what antigens are self-antigens versus foreign antigens. The MHC II transactivator (*Ciita*) controls the transcription of the MHC II heterodimer (*H2-Aa*, *H2-Ab1*) to form a complex with invariant chain (*Cd74*) (64–66). The MHC II/invariant chain complex is transported to the endosomal/lysosomal compartments, where cathepsin S (*Ctss*) and other proteases degrade the invariant chain. Upon invariant chain degradation, the chaperone HLA-DM (*H2-Dma*) promotes high-affinity peptide loading onto the MHC II complex (67, 68). The stable MHC II–peptide complex is then transported to the cell surface for CD4^+^ T cell detection. Once this complex is recognized by the T cell receptor, costimulatory molecules CD40 and/or CD80/86 are activated to promote T cell activation and proliferation (69–71).

Therefore, MHC II antigen presentation genes were assessed in BM via nCounter analysis (Figure 6, A and B, and Supplemental Figure 2). Gene variants of the class II complex (*H2-Aa*, *H2-Ab1*) and invariant chain (*Cd74*) were not altered in alveolar BM of SPF versus GF mice (Figure 6A) or saline versus CHX mice (Figure 6B). *H2-Dma* and *Ctss* were increased in the alveolar BM of SPF versus GF mice (Figure 6A) and saline versus CHX mice (Figure 6B). *Ciita*, *Cd40*, and *Cd86* were expressed at higher levels in the alveolar BM of SPF versus GF mice (Figure 6A), while only *Cd86* was increased in the alveolar BM of saline versus CHX mice (Figure 6B).

A lesser known promoter of MHC II signaling, IL-16 is a proinflammatory cytokine that initiates CD4^+^ T cell chemotaxis (72, 73) and promotes the expression and activity of MHC II components (74, 75). *Il16* was upregulated in alveolar BM of SPF versus GF mice (Figure 6A) and saline versus CHX mice (Figure 6B). Conversely, MHC II–associated genes were similar within the long BM of SPF versus GF mice (Figure 6A) and saline versus CHX mice (Figure 6B).

To corroborate the alterations in MHC II gene expression, flow cytometric analysis was performed to evaluate professional antigen-presenting cells, such as B cells (Supplemental Figure 3), macrophages (Supplemental Figure 4), and DCs (Figure 6, C–H). There were no differences in activated B cells (Supplemental Figure 3), M1 macrophages (Supplemental Figure 4, C–F), and M2 macrophages (Supplemental Figure 4, G–J) in alveolar BM or long BM of SPF versus GF mice or saline versus CHX mice. Interestingly, the frequency of activated classical DCs was enhanced in the alveolar BM, but not long BM, of SPF versus GF mice (Figure 6, E and F) and saline versus CHX (Figure 6, G and H).

### Commensal oral microbiota promotes CD4^+^ T cell activation in alveolar BM.

Understanding that MHC II–restricted antigen presentation is required for CD4^+^ Th cell–dependent immune responses (69–71), flow cytometry was utilized to evaluate alterations in naive (CD45^+^CD3^+^CD4^+^CD8^–^CD62L^+^CD44^–^), memory (CD45^+^CD3^+^CD4^+^CD8^–^CD62L^+/–^CD44^+^), and activated (CD45^+^CD3^+^CD4^+^CD8^–^CD28^+^) CD4^+^ Th cells (Figure 7 and Supplemental Figure 5).

Naive CD4^+^ Th cells (Figure 7, C–F) and activated CD4^+^ Th cells (Figure 7, G–J) were similar in long BM of SPF versus GF mice and saline versus CHX mice. Conversely, naive CD4^+^ Th cells were suppressed in alveolar BM of both SPF versus GF mice (Figure 7, C and D) and saline versus CHX mice (Figure 7, E and F). Furthermore, activated CD4^+^ Th cells were upregulated in alveolar BM of SPF versus GF mice (Figure 7, G and H) and saline versus CHX mice (Figure 7, I and J). Moreover, central and effector memory CD4^+^ T cells were assessed in alveolar and long BM of SPF versus GF mice and saline versus CHX mice (Supplemental Figure 5). While central memory CD4^+^ T cells were similar in oral and nonoral BM of SPF versus GF mice and saline versus CHX mice, effector memory CD4^+^ T cells were increased in long BM of SPF versus GF mice. The increased frequency of activated CD4^+^ Th cells observed in the alveolar BM of SPF versus GF mice (Figure 7, G and H) and saline versus CHX mice (Figure 7, I and J) importantly supports the enhanced MHC II antigen presentation phenotype found in the alveolar BM of SPF mice (Figure 6).

### Commensal oral microbiota promotes activation of alveolar BM–derived DCs in vitro.

In vitro studies were executed to validate the MHC II–mediated DC (Figure 6) and CD4^+^ T cell activation phenotype (Figure 7) detected in alveolar BM of SPF versus GF mice and saline versus CHX mice. DCs derived from alveolar BM and long BM were allostimulated with splenic CD4^+^ T cells for 3 days. Culture supernatants were collected and assessed for the presence of IL-2, a T cell activation and proliferation marker. IL-2 expression was significantly decreased in SPF versus GF alveolar BM cultures (Figure 8A) and trending downward in saline versus CHX alveolar BM cultures (Figure 8B). IL-2 was similar in supernatants from SPF versus GF (Figure 8A) and saline versus CHX long BM cultures (Figure 8B). To corroborate IL-2 findings, DCs and T cells from the allostimulation assay were evaluated for activation markers via flow cytometry (Figure 8, C and D). Alveolar BM–derived DC activation was elevated in SPF versus GF (Figure 8, E and F) and saline versus CHX cultures (Figure 8, G and H). Furthermore, T cell activation was significantly increased in SPF versus GF alveolar BM cultures (Figure 8, I and J) and trended upward in saline versus CHX alveolar BM cultures (Figure 8, K and L). Findings from long BM cultures derived from SPF versus GF mice (Figure 8, F and J) and saline versus CHX mice (Figure 8, H and L) were consistent with in vivo results showing no differences in DC (Figure 6, E–H) and T cell activation (Figure 7, G–J). Considering that CD4^+^ Th cell activation supports osteoclastogenesis through both RANKL-dependent and -independent mechanisms (76–78), our findings suggest that commensal oral microbiota has catabolic osteoimmunomodulatory effects on alveolar bone that are mediated in part by MHC II antigen presentation processes.

### Commensal oral microbiota upregulates proinflammatory CD4^+^ T cell–mediated immunity in alveolar BM.

Previous studies have shown that CD4^+^ helper subsets, Th1 cells and Th17 cells (79–83), have pro-osteoclastic actions that promote inflammatory alveolar bone destruction. Conversely, other CD4^+^ Th subsets, Tregs and Th2 cells, may attenuate alveolar bone loss (84–86). Therefore, CD4^+^ T cell subsets were analyzed in alveolar and long BM by flow cytometry (Figure 9 and Supplemental Figure 6). Th1 (CD3^+^CD4^+^CD8^–^CD183^+^T-bet^+^) cells were increased in alveolar BM of SPF versus GF mice (Figure 9, E and F), and Th17 (CD3^+^CD4^+^CD196^+^RORγt^+^) cells were upregulated in alveolar BM of both SPF versus GF mice (Figure 9, I and J) and saline versus CHX mice (Figure 9, K and L). Th2 (CD3^+^CD4^+^GATA3^+^IRF4^–^) cells were decreased in the alveolar BM of SPF versus GF mice (Supplemental Figure 6, E and F), while Tregs (CD3^+^CD4^+^CD8^–^CD25^+^FoxP3^+^) displayed no differences in alveolar BM of either SPF versus GF mice or saline versus CHX mice (Supplemental Figure 6, I–L). To the contrary, CD4^+^ Th cell subsets were similar within the long BM of SPF versus GF mice and saline versus CHX mice (Figure 9 and Supplemental Figure 6).

## Discussion

This investigation of commensal microbiota osteoimmune response effects in the BM at oral and nonoral skeletal sites intriguingly reveals that the commensal oral microbiota has unique immunostimulatory effects that drive the induction of TLR and MHC II–mediated immunity in alveolar BM. Prior research has discerned that commensal microbiota–derived MAMPs transit epithelial barriers at low levels in health, which leads to PRR signaling modulation of the BM hematopoietic compartment (1, 53–55). The upregulation of MYD88-dependent TLR-signaling in alveolar BM (but not long BM), reported herein, supports the notion that commensal oral microbiota–derived ligands pass through oral epithelial barriers at appreciable levels in health to stimulate marrow hematopoiesis in the alveolar bone complex.

Unlike other epithelial tissues that act as an impermeable barrier to colonizing microbiota communities, the junctional epithelial attachment at the tooth surface is highly permeable (25–27). While whole microbes do not typically penetrate the junctional epithelium, research has delineated that microbial byproducts pass through this highly permeable epithelium in both health and disease. Bacteria-derived MAMPs and foreign antigens topically applied to the intact healthy junctional epithelium have been shown to penetrate the epithelium, infiltrate the gingival connective tissue (28–31), reach the ABC (32, 33), and even enter the alveolar BM (34). Histological evaluation of radiolabeled antigen distribution within periodontal tissue sections suggest that foreign antigens reach the alveolar bone complex not by simple diffusion, but rather via lymphatic vessels. Whereas lymphatic vessels are not characteristically present in long bone, research has discerned that lymphatic vessels are a normal constituent of the alveolar bone complex in the healthy periodontium (35–38), acting as “a vehicle for lymphatic drainage of teeth and periodontal membranes” (37).

Knowing that oral microbiota–derived ligands/byproducts penetrate the junctional epithelium to reach the alveolar bone complex in health (32–34), and that the gingival lymphatic extension from the junctional epithelium to the alveolar bone is a normal anatomic phenomenon (35–38), it intriguingly appears that alveolar bone is continuously exposed to oral microbiota–derived MAMPs and antigens through dentoalveolar lymphatics. With constant antigen exposure in the alveolar lymphatic channels, the comparison of antigen presentation processes at oral and nonoral skeletal sites notably demonstrates that the commensal oral microbiota has distinct immunoregulatory actions that induce MHC II antigen–restricted presentation in alveolar BM. Interestingly, *Il16* expression was increased in alveolar BM but not long BM of SPF versus GF mice. While few studies have identified IL-16 as a chemotactic cytokine for CD4^+^ T cells and Th1 cells (72, 73), other reports have linked IL-16 to enhanced osteoclastogenesis (87) and periopathogenesis (88–90). However, the role of IL-16 and specific MHC II components in alveolar bone health is unknown. Further studies are needed to elucidate the role of antigen processing and presentation in the alveolar BM complex.

The adult BM is the primary site for hematopoietic stem cells (HSCs) (91–93). Extensive research has discerned that HSC niches in long BM are endosteal and/or perivascular in nature (91–94). Endosteal cells and perivascular cells produce local factors in the long BM environment that impact HSC maintenance and regulate the function of downstream hematopoietic cell populations (91–94), and this ultimately has implications for skeletal remodeling and homeostasis (94). Findings from the current report support the notion that the HSC niche housed within alveolar BM is distinct from nonoral skeletal sites. Specifically, it appears that the gingival lymphatic extension from the junctional epithelium to alveolar bone provides a unique HSC niche in the alveolar BM. Ongoing research is necessary to discern the role of the alveolar BM-lymphatic-HSC niche in oral microbiota — host immune response mechanisms regulating periodontal health and disease.

The burden of the commensal oral microbiota has been shown to drive naturally occurring alveolar bone loss in health (13, 40–42). The 0.12% CHX oral rinse studies carried out in SPF mice demonstrated that local antiseptic depletion of the commensal oral microbiota reduces naturally occurring alveolar bone loss. Clinical and preclinical reports have revealed that CHX treatments suppress the oral microbiome (20–22) and protect against periodontitis-induced alveolar bone destruction (18, 19). To our knowledge, this is the first investigation showing that CHX oral rinses attenuate naturally occurring alveolar bone loss. The current findings suggest that oral antiseptic rinses could be employed to support alveolar bone homeostasis in the healthy periodontium.

## Methods

### Mice.

Male GF C57BL/6T mice were acquired from the MUSC Gnotobiotic Facility. Male SPF C57BL/6T mice were purchased from Taconic and housed in ventilated cages in an SPF vivarium. Mice were euthanized at age 12–13 weeks for studies comparing SPF and GF mice. In the oral rinse model, male SPF C57BL/6T mice were administered twice daily oral rinses of saline-control (1× PBS) or 0.12% chlorhexidine gluconate (ESPE Peridex; 48878-0620-1; 3M Company) from age 8 to 12 weeks or from age 6 to 12 weeks. Oral rinses were performed by taking up 150 μL of saline or chlorhexidine into a LASIK Expanded Spear sponge (581709; Beaver-Visitec International), which was inserted into the murine oral cavity for 30 seconds (Supplemental Video 1). Oral rinses were performed every 12 hours, and animals were euthanized 12 hours following the final oral rinse. Room temperature and humidity were maintained within recommended ranges of the NIH *Guide for the Care and Use of Laboratory Animals* (National Academies Press, 2011).

### μCT.

Tibiae, vertebrae, mandibles, and maxillae were fixed in 10% neutral-buffered formalin (NBF). Tibiae and vertebrae were scanned with Scanco μCT 40 Scanner (Scanco Medical): x-ray tube potential = 55 kVp; integration time = 200 ms; voxel size = 6 μm^3^. Mandible and maxilla specimens were scanned with Scanco μCT 40 Scanner: x-ray tube potential = 70 kVp; integration time = 200 ms; voxel size = 10 μm^3^. Calibrated 3-dimensional images were reconstructed. L5 vertebral bodies and tibiae were evaluated using Analyze 12.0 Bone Microarchitecture Analysis software (AnalyzeDirect). For vertebral trabecular analysis, axial CT slices were analyzed beginning 150 μm coronal to the caudal epiphyseal growth plate and extending 500 μm coronally (threshold = 1750 Hounsfield Units [HU]). For tibia cortical analysis, transverse CT slices were analyzed in a 1000 μm segment of the tibia mid-diaphysis at a threshold of 2500 HU. μCT analyses for maxillae and mandibles were performed using AnalyzePro Segment and Measure Evaluation Program (AnalyzeDirect). Trabecular bone parameters were evaluated in the maxillary first molar trifurcation by creating a volume of interest (VOI) defined by linearly morphing 2 consecutive cylinder ROIs (Figure 3A). The initial ROI began 100 μm apical to the fornix/roof of the furcation, and the second ROI was 200 μm apical to the initial ROI. Cortical thickness was evaluated in a 200 μm mesial-distal ROI in the lingual and buccal cortical plate of the mandibular first molar bifurcation. ROI was centered at the midpoint between the mesial and distal roots in the sagittal plane; measurements were then performed from 10 μm to 100 μm in 10 μm increments distal and mesial to the midpoint (Figure 3F; maxilla and mandible threshold = 270 HU). Data are reported in accordance with standardized nomenclature (95).

Alveolar bone loss was determined by measuring the linear distance from the CEJ to the ABC at the mesiobuccal line angle, distobuccal line angle, and mid-lingual aspect of the maxillary first molar. The mid buccal-lingual aspect of the molar was determined, and the CEJ at the mesial and distal aspect of the tooth were aligned at 0° in the sagittal plane. The coronal height of contour was identified at the mesiobuccal line angle, the distobuccal line angle, and the midlingual aspect of the molar in the axial plane. These landmarks were used as the midpoint for performing CEJ to ABC linear measurements. Initial measurement was performed at the midpoint, and measurements were then carried out 10, 20, and 30 μm distal and mesial to the midpoint.

### Histomorphometry.

Tibiae, maxillae, and mandibles were fixed in 10% NBF, decalcified in 14% EDTA, and processed for paraffin histology, as previously described (14, 96). Serial sections (5 μm) were acquired from frontal sections of proximal tibia and mandibular first molar and sagittal sections of maxillary first molar. H&E sections were analyzed for inflammatory cell infiltrate in the lingual and buccal junctional epithelium of the mandibular first molar, quantified as cell area per tissue area. Tibia and maxilla sections were TRAP stained (Fast Green counterstained) for histomorphometric analysis of osteoclasts; TRAP^+^ multinucleated (≥3 nuclei) cells lining trabecular bone were scored as osteoclasts. ROI in proximal tibiae sections was carried out as previously described (14, 96). ROI in maxillae sections was assigned to the alveolar bone in the first molar trifurcation. Images were acquired at 200× via Olympus BX61 microscope (Olympus America Inc.) and analyzed using Visiopharm software. Data are reported in accordance with standardized nomenclature (97), as previously described (13, 14, 96).

### Immunofluorescence.

Immunofluorescence staining was performed in paraffin-embedded tissue sections, as previously described (96). LYVE-1 (Abcam) staining was performed on maxilla sagittal sections. Images were acquired via an Olympus BX-61 fluorescence microscope with Olympus cellSens software.

### Quantitative PCR (qPCR) mRNA analysis.

Mandibular gingival RNA was isolated by the TRIzol Reagent (Invitrogen) method. Total RNA was quantified via NanoDrop 1000 (Thermo Fisher Scientific). cDNA was generated using TaqMan Random Hexamers and Reverse Transcription Reagents (Applied Biosystems) and amplified by TaqMan primers/probes and Universal PCR Master Mix by the StepOnePlus System (Applied Biosystems). *Rn18s* was utilized as an internal control gene; relative quantification of data was carried out by the comparative Ct method (2^–ΔΔCt^) (98), as previously described (14, 96).

### qPCR 16S rDNA analysis.

Gingiva and fecal pellets were collected at euthanasia, and bacterial DNA was isolated using the Qiagen DNeasy Powersoil Pro Kit, per manufacturer’s guidelines (Qiagen). qPCR reactions were carried out on the StepOnePlus System (Applied Biosystems) for 30 cycles (96, 99, 100). Bacterial load was determined by normalizing the Universal 16S gene to a bacterial DNA standard (ZymoBIOMICS, Zymo Research), as previously described (100). Relative quantification of Universal 16S data was performed by the 2^–ΔCt^ method (101, 102). Phylum level outcomes are reported relative to the Universal 16S gene; relative quantification by the 2^–ΔΔCt^ method (100). Forward (F)/reverse (R) primer sequences included (Integrated DNA Technologies): Universal 16S (99): F, 5′-ACT CCT ACG GGA GGC AGC AGT-3′; R, 5′-ATT ACC GCG GCT GGC-3′. Proteobacteria (99): F, 5′-TCGTCAGCTCGTGTYGTGA-3′; R, 5′-CGTAAGGGCCATGATG-3′. Actinobacteria (99): F, 5′-TACGGCCGCAAGGCTA-3′; R, 5′-TCRTCCCCACCTTCCTCCG-3′. Bacteroidetes (99): F, 5′-CRAACAGGATTAGATACCCT-3′; R, 5′-GGTAAGGTTCCTCGCGTAT-3′. Firmicutes (99): F, 5′-TGAAACTYAAAGGAATTGACG-3′; R, 5′-ACCATGCACCTGTC-3′. Fusobacteria (103): F, 5′-C(A/T)AACGCGATAAGTAATC-3′; R, 5′-TGGTAACATACGA(A/T)AGGG-3′. Spirochaetes (103): F, 5′-GAGAGTTTGATYMTGGCTCAG-3′; R, 5′-GTTACGACTTCACCCTCCT-3′.

### Alveolar BM isolation.

Alveolar BM was isolated in the mandible marrow space, which was accessed by rotating a 28 G needle through the lateral surface of the ramus. BM was flushed from the ascending ramus of the left and right hemisected mandible and processed for nCounter gene expression, flow cytometry, or in vitro studies.

### nCounter gene expression.

Alveolar (mandible) and long bone (tibia and femur) marrow were flushed with TRIzol Reagent; RNA extracted following the manufacturer’s protocol. Total RNA was quantified by NanoDrop 1000. nCounter Mouse PanCancer Panel (NanoString Technologies) was utilized. The nCounter gene expression system is a multiplexed probe detection system, where a probe library is constructed with 2 sequence-specific probes for each gene of interest (104, 105). Hybridization of samples was performed, and products were run on the nCounter preparation station, following manufacturer’s protocol. Data were analyzed via nSolver Analysis Software v2.6 (NanoString Technologies), normalized to the geometric means of spiked-in positive controls, negative controls, and built-in internal control genes. Data were reported as fold change or as normalized mRNA counts, as described previously (14, 96).

### Flow cytometry.

Long BM (femur), alveolar BM (mandible), and CLN cells were isolated, washed, and labeled for analysis, as previously reported (14, 96). Briefly, live cells were treated with FcR-block (Miltenyi Biotec) and labeled for cell surface markers. Antibody information is included in Supplemental Table 1. Intracellular staining was performed after FcR-block treatment and cell surface staining, where cells were treated with fixation/permeabilization buffer (eBioscience) and labeled for intracellular transcription factors (Supplemental Table 1). Dead cells were removed from analysis by propidium iodide (Miltenyi Biotec) or Live/Dead Fixable Yellow dead cell kit (Invitrogen). Fluorescence minus one (FMO) controls for T-bet and RORγt cellular staining are included in Supplemental Figure 7. A minimum of 10,000 live gated cells were gated on LIVE/DEAD Fixable Dead Cell stain kit (Thermo Fisher Scientific; L34967) and were analyzed for each specimen. Data were acquired by MACSQuant System (Miltenyi Biotec) for CLN cells and by the LSR Fortessa System (BD Biosciences) for long bone and alveolar BM cells. Data were analyzed by FlowJo 11.0 software (Tree Star Inc.).

### DC/T cell allostimulation assay.

Upon euthanasia (day 0), long BM and alveolar BM were flushed, plated, and cultured with complete RPMI media (10% FBS, 1% penicillin-streptomycin) with 50 μM β-Mercaptoethanol (β-ME) and 20 ng/mL GM-CSF (R&D Systems), as described (106). Cells were cultured with 20 ng/mL GM-CSF for 3 days to differentiate into DCs; media was changed every other day. Beginning on day 4, cells were then treated with 20 ng/mL GM-CSF and 5 ng/mL IL-4 (R&D Systems) for 2 days, as described (106). On Day 6, alveolar BM and long BM cells were incubated with Pan DC microbeads (Miltenyi Biotec), and AutoMACS Sorter (Miltenyi Biotec) was employed to separate DCs. DCs were washed, counted, and plated for allostimulation assay at 5.0 × 10^5^ cells/mL in 96-well round-bottom plates (Corning) in triplicate. Simultaneously, spleens from the same animals were harvested on day 0 and placed in Tissue Storage Solution (Miltenyi Biotec) for 24 hours. On Day 1, spleens were dissociated through a 40 μm strainer, and cells were washed and incubated with CD4^+^ T cell Biotin-antibody cocktail microbeads (Miltenyi Biotec). AutoMACS Sorter (Miltenyi Biotec) was used to separate CD4^+^ T cells. T cells were then cultured in complete RPMI media with 50 ng/mL IL-2 for 5 days; media were refreshed every other day. On day 6, CD4^+^ T cells were collected, counted, and plated at a 4:1 ratio with either alveolar bone–derived or long bone–derived DCs from the same animal. DC/T cell cocultures were incubated for 3 days in complete RPMI media with 50 μM β-ME. Cells were then collected and stained for activated DCs and CD4^+^ T cells (Supplemental Table 1) and analyzed by flow cytometry. A minimum of 10,000 live gated cells were gated on LIVE/DEAD Fixable Dead Cell stain kit (Thermo Fisher Scientific; L34967) and were analyzed for each specimen. Data were acquired by LSR Fortessa System (BD Biosciences) and analyzed by FlowJo 11.0 software (Tree Star Inc.).

### ELISA analysis.

Culture supernatants were collected from DC/T cell allostimulation assay cocultures on day 3 and stored at –80°C. Quantikine IL-2 ELISA kit (R&D Systems) was used following manufacturer’s guidelines. All reactions were performed in triplicate.

### Statistics.

Unpaired 2-tailed *t* tests were performed comparing the same cell/tissue isolate from alveolar bone in SPF versus GF mice and saline versus CHX mice. Unpaired 2-tailed *t* tests were performed comparing the same cell/tissue isolate from nonoral skeletal sites in SPF versus GF mice and saline versus CHX mice (GraphPad Prism 8.7; GraphPad Software Inc.). Data are reported as mean ± SEM, and significance is designated as *P < 0.05, **P < 0.01, and ***P < 0.001. Power analysis was performed in consultation with the MUSC Bioinformatics Core and was based on the authors’ prior experience.

### Study approval.

Research was approved by the MUSC Animal Protocols Review Board and was performed in accordance with the NIH *Guide for the Care and Use of Laboratory Animals* (National Academies Press, 2011) and reported by the ARRIVE guidelines.

## Author contributions

Study conception was contributed by CMN. Study design was contributed by JDHS, JDA, RPD, CW, and CMN. Data acquisition was contributed by JDHS, JDA, NAP, MBK, BEM, MEC, EH, and CMN. Data analysis was contributed by JDHS, JDA, NAP, MBK, BEM, MEC, EH, and CMN. Data interpretation was contributed by JDHS, JDA, RPD, CW, and CMN. The manuscript was drafted by JDHS, JDA, and CMN. All authors approved the final version of the manuscript. JDHS and CMN take responsibility for the integrity of the data analysis.

## Supplementary Material

Supplemental data

Supplemental video 1

## Version 1 01/25/2022

In-Press Preview

## Version 2 02/22/2022

Electronic publication
