# Supplementary material for: Commensal oral microbiota induces osteoimmunomodulatory effects separate from systemic microbiome in mice
Source: JCI Insight. 2022 Feb 22;7(4):e140738. doi: 10.1172/jci.insight.140738 (PMC8876522; doi:10.1172/jci.insight.140738)
Supplement: Supplemental data [file jciinsight-7-140738-s165.pdf]

Supplemental Table 1: Flow Cytometry Antibodies					
**Cell Type**	**Tissue**	**Cell Marker**	**Fluorescent Tag**	**Vendor**	**Clone**
Monocytes / Neutrophils	CLNs	CD11b	APC	Miltenyi	M1/70.15.11.5
		Ly6G	PacB	Biolegend	18A
		Ly6C	FITC	Novus Bio.	HK1.4
		F4/80	PE	eBioscience	BM8
Macrophages (M1s, M2s)	CLNs	CD11b	APC	Miltenyi	REA592
		CD68	PE-Vio770	Miltenyi	REA835
		MHC II	FITC	Miltenyi	REA528
		CD64	PE	eBioscience	MR6F3
		CD206	APC-Vio770	Miltenyi	REA286
T-cells	ABM, LBM	CD3	APC-Vio770	Miltenyi	REA641
		CD4	VioBlue	Miltenyi	REA604
		CD8	VioGreen	Miltenyi	REA601
		CD62L	PerCP-Cy5.5	Thermofisher	MEL-14
		CD45	AF700	Miltenyi	30-F11
		CD44	FITC	Miltenyi	REA664
		CD28	PE	Miltenyi	REA806
Dendritic cells	ABM, LBM	CD45	AF700	Thermofisher	30-F11
		CD11b	PE-Vio770	Miltenyi	REA592
		CD11c	VioBlue	Miltenyi	REA754
		B220	PerCP-Cy5.5	Thermofisher	RA3-6B2
		CD8	APC-Vio770	Miltenyi	REA601

		MHC II	VioGreen	Miltenyi	REA813
		CD86	APC	Miltenyi	REA1190
		Siglec-H	FITC	Miltenyi	REA819
B-cells	ABM, LBM	CD45	PE-Vio770	Miltenyi	REA737
		CD19	VioBlue	Miltenyi	REA749
		B220	PerCP-Cy5.5	Thermofisher	RA3-6B2
		MHC II	VioGreen	Miltenyi	REA813
		IgM	FITC	Miltenyi	REA979
		CD3	APC-Vio770	Miltenyi	REA641
		CD86	APC	Miltenyi	REA1190
Macrophages	ABM, LBM	CD45	APC-Vio770	Miltenyi	REA737
(M1s, M2s)		CD11b	PE-Vio770	Miltenyi	REA592
		CD11c	VioBlue	Miltenyi	REA754
		F4/80	PE-Cy5	Thermofisher	BM8
		MHC II	VioGreen	Miltenyi	REA813
		CD64	PE	Miltenyi	REA286
		CD86	APC	Miltenyi	REA1190
		CD206	AF700	Thermofisher	MR6F3
T_H1 cells	ABM, LBM	CD3	APC-Vio770	Miltenyi	REA641
		CD4	VioBlue	Miltenyi	REA604
		CD8	VioGreen	Miltenyi	REA601
		CD183	FITC	Miltenyi	REA724
		Tbet	APC	Miltenyi	REA102
T_H17 cells	ABM, LBM	CD3	APC-Vio770	Miltenyi	REA641

		CD4	VioBlue	Miltenyi	REA604
		CD196	PE	Miltenyi	REA227
		RORgT	APC	Miltenyi	REA278
T_H2 cells	ABM, LBM	CD3	APC-Vio770	Miltenyi	REA641
		CD4	VioBlue	Miltenyi	REA604
		GATA3	AF700	Thermofisher	TWAJ
		IRF4	PE-Vio770	Miltenyi	REA201
T_{REG} cells	ABM, LBM	CD3	APC-Vio770	Miltenyi	REA641
		CD4	VioBlue	Miltenyi	REA604
		CD8	VioGreen	Miltenyi	REA601
		CD25	PE-Vio770	Miltenyi	REA570
		FoxP3	PE	Miltenyi	REA788
Dendritic cells	In vitro studies	CD11c	VioBlue	Miltenyi	REA754
		MHC II	VioGreen	Miltenyi	REA813
		CD86	APC	Miltenyi	REA1190
T-cells	In vitro studies	CD3	APC-Vio770	Miltenyi	REA641
		CD4	VioBlue	Miltenyi	REA604
		CD28	PE	Miltenyi	REA806
Live/Dead fixable stain	All studies	Live/dead	Qdot605	Thermofisher	N/A

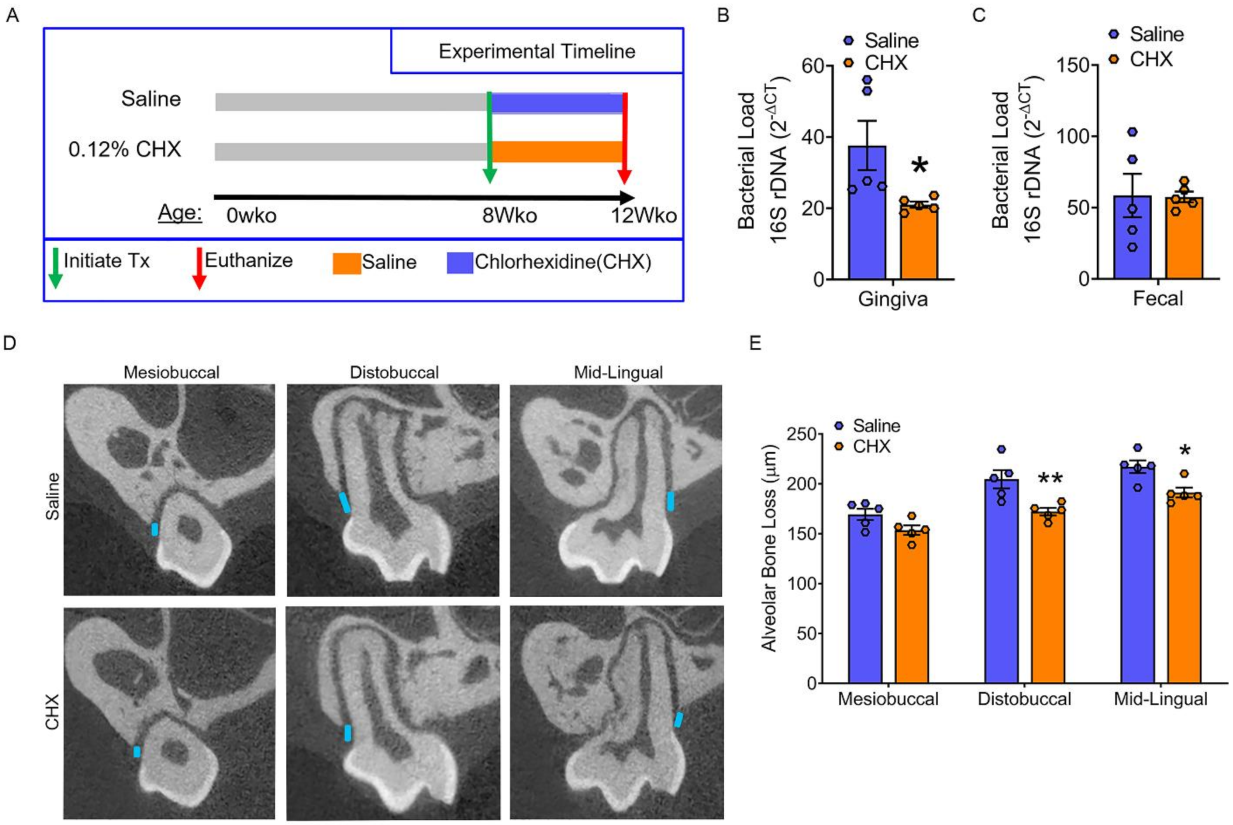

Supplemental Figure 1. Four-week treatment with CHX oral rinse suppresses commensal oral microbiota and protects from alveolar bone loss. (A) Experimental timeline of SPF mice treated with CHX vs. Saline oral rinse from age 8 to 12 weeks. **(B-C)** 16S rDNA analysis of bacterial load for **(B)** maxillary gingiva and **(C)** fecal pellets; data reported as $2^{-\Delta C_T}$; n=5/gp. **(D-E)** Alveolar bone loss was assessed by evaluating the linear distance between the cementoenamel junction (CEJ) and alveolar bone crest (ABC) at the maxillary first molar in reconstructed micro-CT images. **(D)** Representative micro-CT images displaying CEJ-ABC linear distance (blue line) at the mesiobuccal, distobuccal, and mid-lingual aspect of the maxillary first molar. **(E)** Quantitative measures of CEJ-ABC linear distance at the mesiobuccal, distobuccal, and mid-lingual aspect of the maxillary first molar; n=5/gp. Unpaired *t*-test; data presented as mean ± SEM; *$p<0.05$, **$p<0.01$.

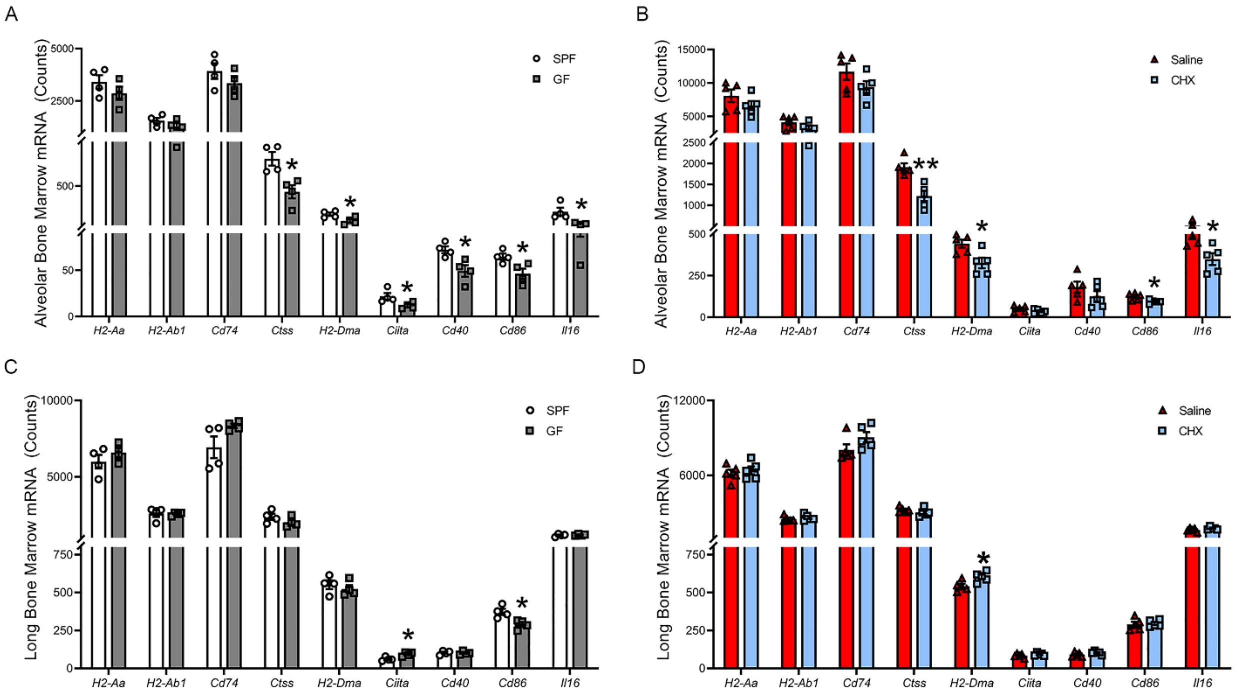

Supplemental Figure 2. Commensal oral microbiota has immunomodulatory effects to induce MHC II antigen presentation genes in alveolar bone marrow. (A-B) nCounter analysis was performed to assess MHC II antigen processing and presentation genes in alveolar bone marrow from **(A)** SPF vs. GF mice (n=4/gp) and **(B)** Saline vs. CHX mice (n=6/gp). Data presented as mRNA counts. **(C-D)** nCounter analysis evaluated MHC II antigen processing and presentation genes in long bone marrow from **(C)** SPF vs. GF mice (n=4/gp) and **(D)** Saline vs. CHX mice (n=6/gp). Data presented as mRNA counts. Unpaired *t*-test; data reported as mean ± SEM; *p<0.05, **p<0.01.

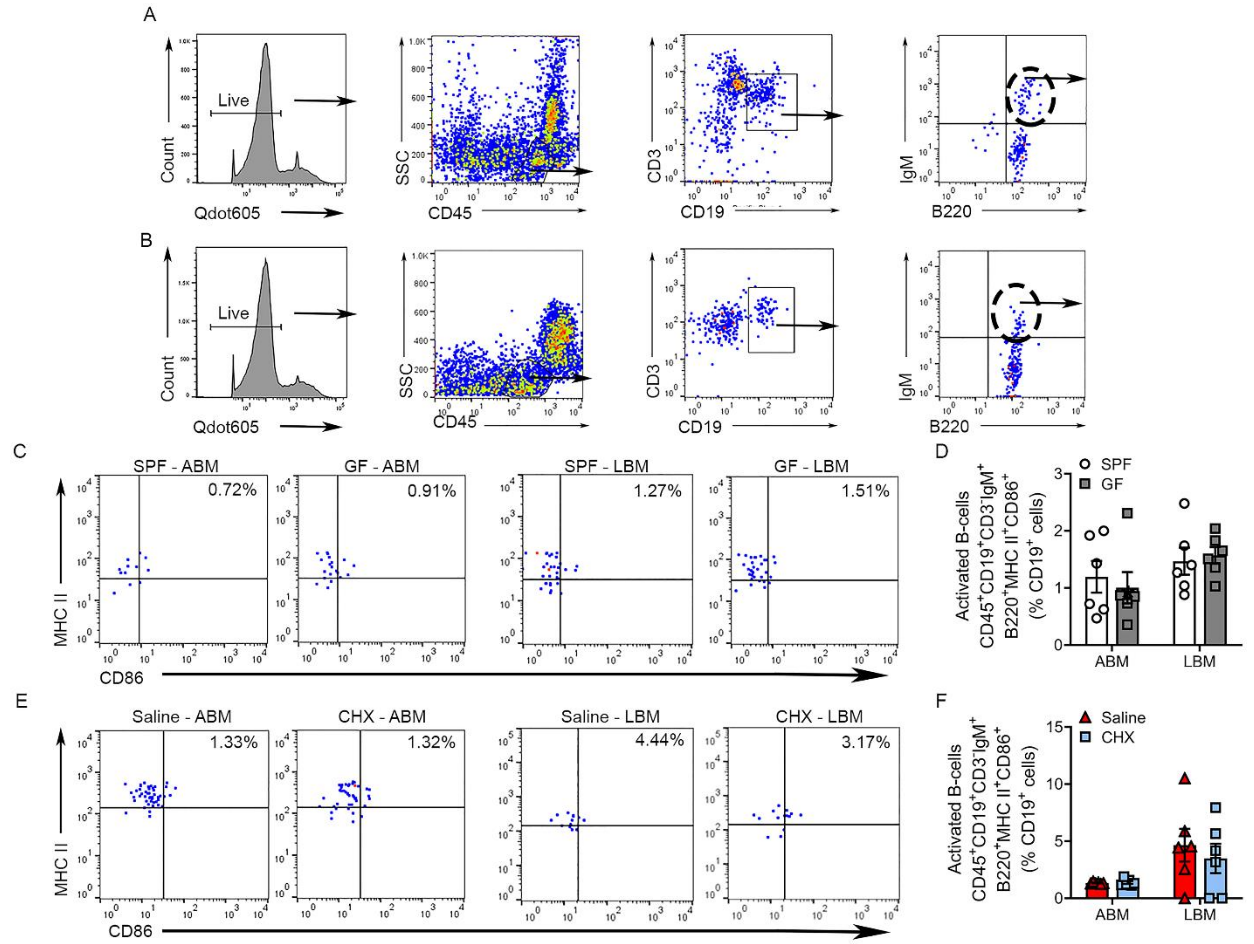

Supplemental Figure 3. Commensal oral microbiota did not alter B-cell activation in alveolar bone marrow. Flow cytometric analysis of activated B-cells in the alveolar bone marrow (ABM) and long bone marrow (LBM), reported as % $CD19^+$ cells; n=6/gp. **(A-B)** Representative gating strategy for activated B-cells in **(A)** ABM and **(B)** LBM. **(C-D)** Representative dot plots and **(D)** quantitation for $CD45^+CD19^+CD3^-IgM^+B220^+$MHC II^+CD86^+ activated B-cells in ABM and LBM of SPF vs. GF mice. **(E)** Representative dot plots and **(F)** quantitation for $CD45^+CD19^+CD3^-$ IgM^+B220^+MHC II^+CD86^+ activated B-cells in ABM and LBM of Saline vs. CHX mice. Unpaired *t*-test; data are presented as mean ± SEM.

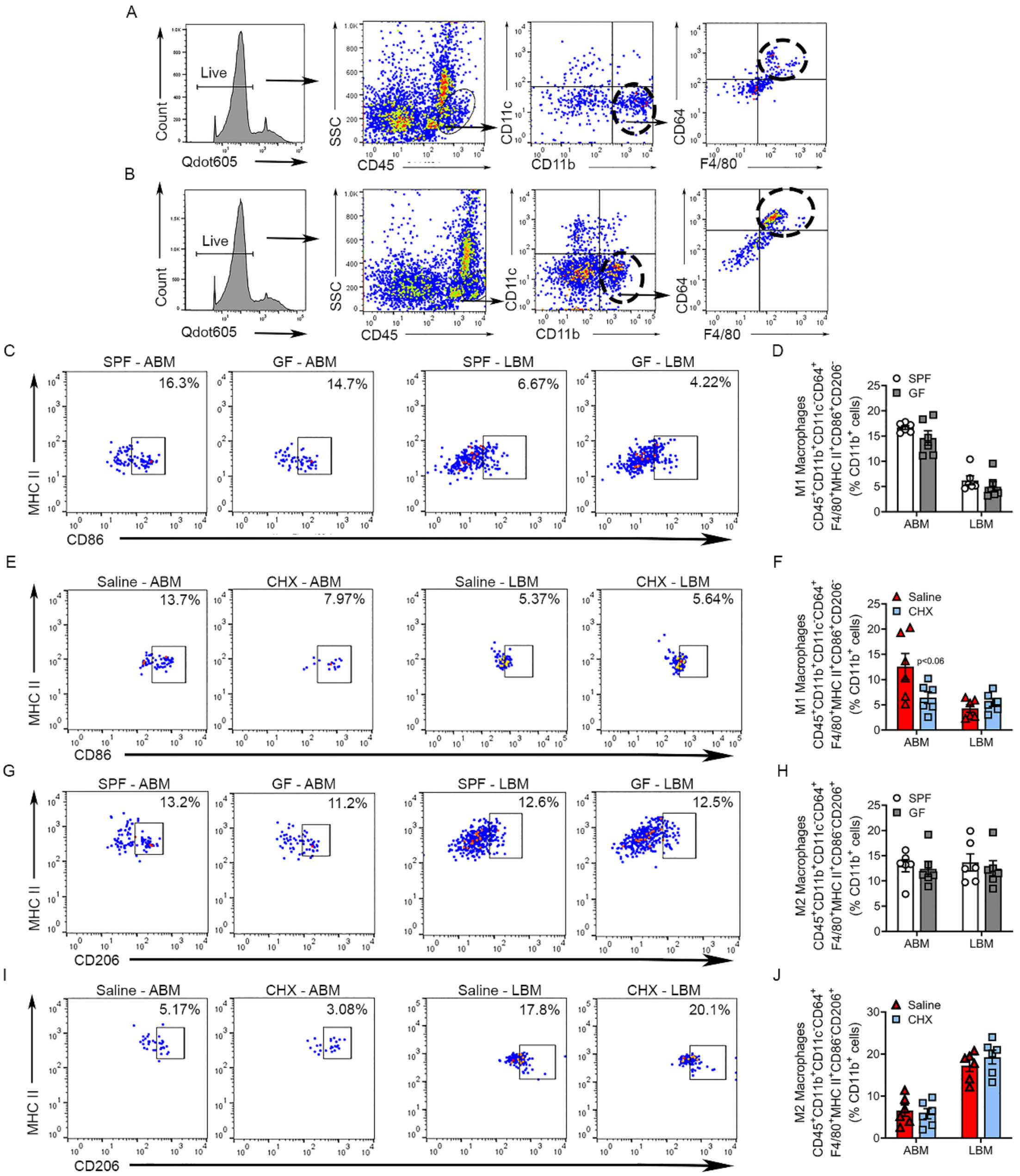

Supplemental Figure 4. Commensal microbiota effects on M1/M2 macrophages in oral and non-oral bone marrow. Flow cytometric analysis of M1 and M2 macrophages in the alveolar bone marrow (ABM) and long bone marrow (LBM), reported as % CD11b$^+$ cells; n=6/gp. **(A-B)** Representative gating strategy for macrophages in **(A)** ABM and **(B)** LBM. **(C)** Representative dot plots and **(D)** quantitation for CD45$^+$CD11b$^+$CD11c$^-$CD64$^+$F4/80$^+$MHC II$^+$CD86$^+$CD206$^-$ M1 macrophages in ABM and LBM of SPF vs. GF mice. **(E)** Representative dot plots and **(F)** quantitation for CD45$^+$CD11b$^+$CD11c$^-$CD64$^+$F4/80$^+$MHC II$^+$CD86$^+$CD206$^-$ M1 macrophages in ABM and LBM of Saline vs. CHX mice. **(G)** Representative dot plots and **(H)** quantitation for CD45$^+$CD11b$^+$CD11c$^-$CD64$^+$F4/80$^+$MHC II$^+$CD86$^-$CD206$^+$ M2 macrophages in ABM and LBM of SPF vs. GF mice. **(I)** Representative dot plots and **(J)** quantitation for CD45$^+$CD11b$^+$CD11c$^-$CD64$^+$F4/80$^+$MHC II$^+$CD86$^-$CD206$^+$ M2 macrophages in ABM and LBM of Saline vs. CHX mice. Unpaired *t*-test; data presented as mean ± SEM.

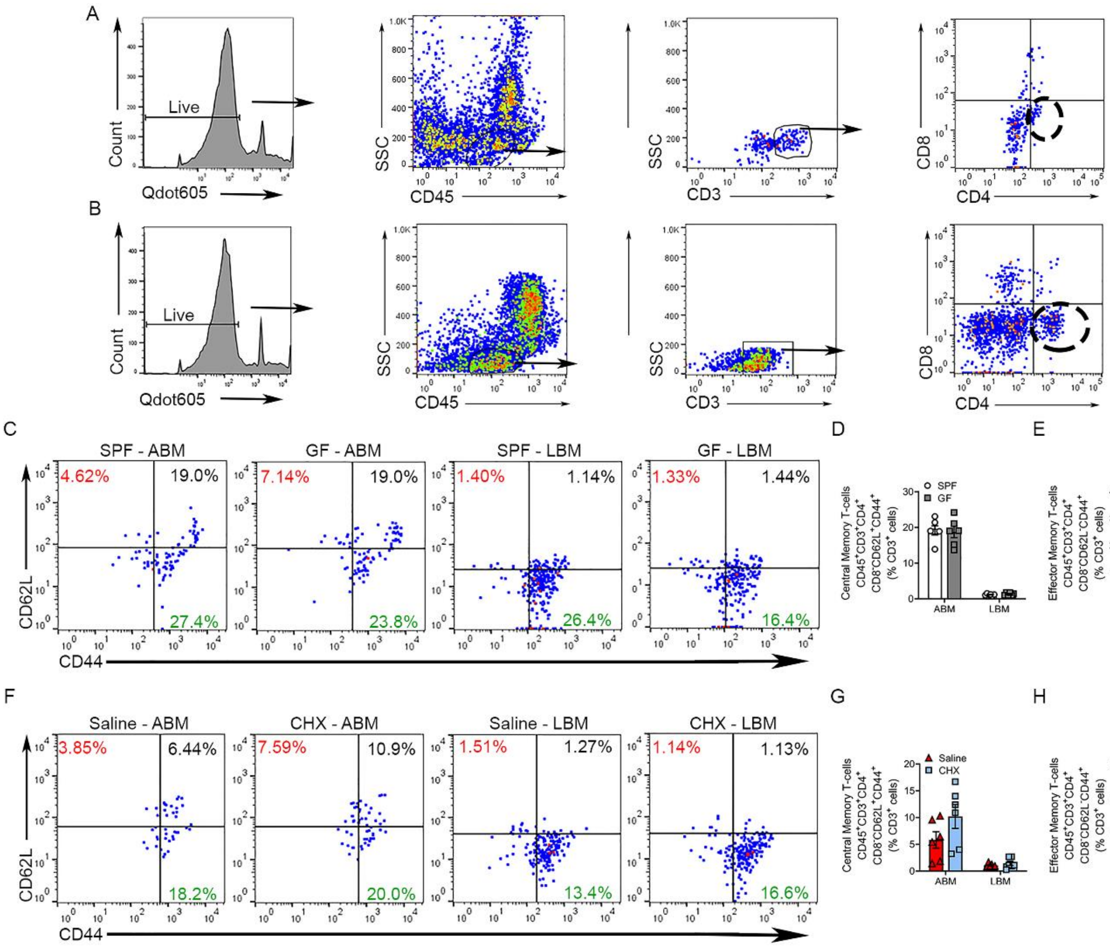

Supplemental Figure 5. Commensal microbiota effects on central and effector memory CD4$^+$ T-cells in oral and non-oral bone marrow. Flow cytometric analysis of CD4$^+$ helper T-cell cells in the alveolar bone marrow (ABM) and long bone marrow (LBM), reported as % CD3$^+$ cells; n=6/gp. (**A-B**) Representative gating strategy for CD4$^+$ T-cells in **(A)** ABM and **(B)** LBM. **(C)** Representative dot plots and quantitation for **(D)** CD45$^+$CD3$^+$CD4$^+$CD8$^-$CD62L$^+$CD44$^+$ central memory T-cells (Upper right; black %) and **(E)** CD45$^+$CD3$^+$CD4$^+$CD8$^-$CD62L$^-$CD44$^+$ effector memory T-cells (Lower right; green %) in ABM and LBM of SPF vs. GF mice. **(F)** Representative dot plots and quantitation for **(G)** CD45$^+$CD3$^+$CD4$^+$CD8$^-$CD62L$^+$CD44$^+$ central memory T-cells (Upper right; black %) and **(H)** CD45$^+$CD3$^+$CD4$^+$CD8$^-$CD62L$^-$CD44$^+$ effector memory T-cells (Lower right; green %) in ABM and LBM of Saline vs. CHX mice. Unpaired *t*-test; data presented as mean ± SEM; ***p<0.001.

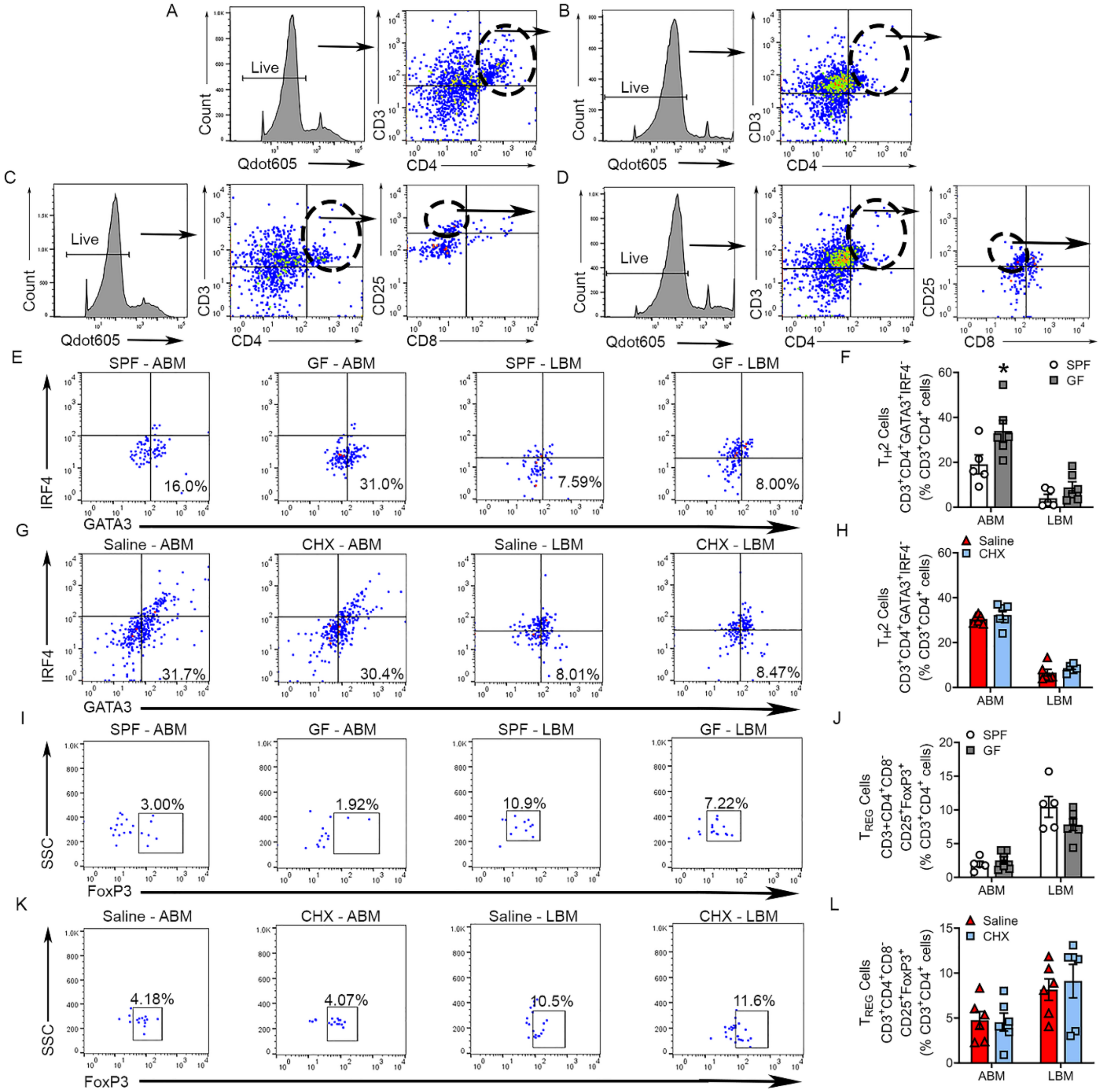

Supplemental Figure 6. Commensal microbiota effects on T_H2 and T_{REG} cells in oral and non-oral bone marrow. Flow cytometric analysis of $CD4^+$ helper T-cell subsets in the alveolar bone marrow (ABM) and long bone marrow (LBM), reported as % $CD3^+CD4^+$ cells; n=5-6/gp. **(A-B)** Representative gating strategy for T_H2 cells in **(A)** ABM and **(B)** LBM. **(C-D)** Representative gating strategy for T_{REG} cells in **(C)** ABM and **(D)** LBM. **(E)** Representative dot plots and **(F)** quantitation for $CD3^+CD4^+GATA3^+IRF4^-$ T_H2 cells in ABM and LBM of SPF vs. GF mice. **(G)** Representative dot plots and **(H)** quantitation for $CD3^+CD4^+GATA3^+IRF4^-$ T_H2 cells in ABM and LBM of Saline vs. CHX mice. **(I)** Representative dot plots and **(J)** quantitation for $CD3^+CD4^+CD8\text{-}CD25^+FoxP3^+$ T_{REG} cells in ABM and LBM of SPF vs. GF mice. **(K)** Representative dot plots and **(L)** quantitation for $CD3^+CD4^+CD8\text{-}CD25^+FoxP3^+$ T_{REG} cells in ABM and LBM of Saline vs. CHX mice. Unpaired *t*-test; data presented as mean ± SEM; *p<0.05.

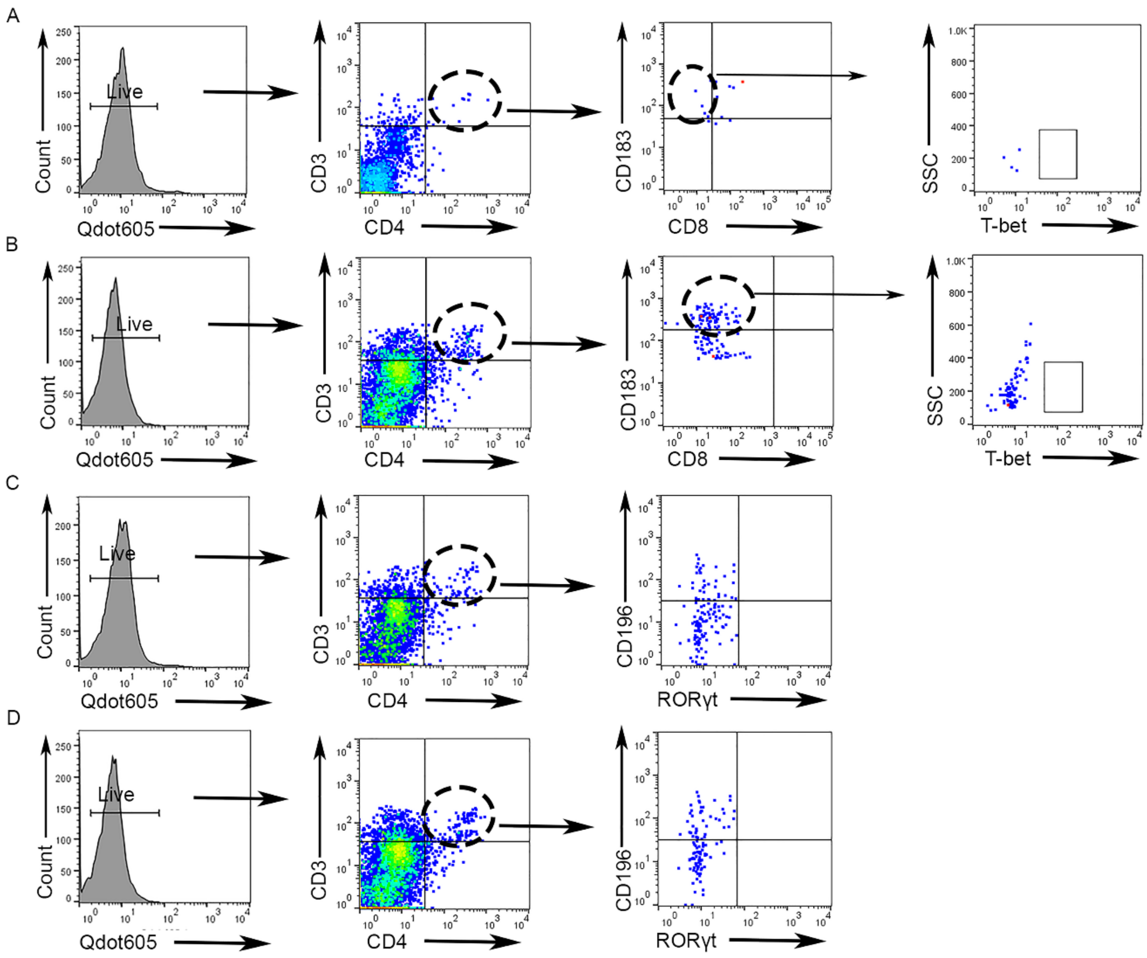

Supplemental Figure 7. Fluorescence Minus One (FMO) controls for T-bet and RORγt. (A-B) Flow cytometric analysis of T_H1 antibody markers without staining for T-bet. Representative gating strategy for T_H1 cells in **(A)** ABM and **(B)** LBM. **(C-D)** Flow cytometric analysis of T_H17 antibody markers without staining for RORγt. Representative gating strategy for T_H17 cells in **(A)** ABM and **(B)** LBM.
